# Plastidic Phosphoglucose Isomerase Is an Important Determinant of Starch Accumulation in Mesophyll Cells, Growth, Photosynthetic Capacity, and Biosynthesis of Plastidic Cytokinins in Arabidopsis

**DOI:** 10.1371/journal.pone.0119641

**Published:** 2015-03-26

**Authors:** Abdellatif Bahaji, Ángela M. Sánchez-López, Nuria De Diego, Francisco J. Muñoz, Edurne Baroja-Fernández, Jun Li, Adriana Ricarte-Bermejo, Marouane Baslam, Iker Aranjuelo, Goizeder Almagro, Jan F. Humplík, Ondřej Novák, Lukáš Spíchal, Karel Doležal, Javier Pozueta-Romero

**Affiliations:** 1 Instituto de Agrobiotecnología (CSIC/UPNA/Gobierno de Navarra), Iruñako etorbidea 123, Mutiloabeti, Nafarroa, 31192, Spain; 2 Department of Chemical Biology and Genetics, Centre of the Region Haná for Biotechnological and Agricultural Research, Faculty of Science, Palacký University, Olomouc, CZ-78371, Czech Republic; 3 Plant Biology and Ecology Department, Science and Technology Faculty, University of the Basque Country, Barrio Sarriena, 48940 Leioa, Spain; 4 Laboratory of Growth Regulators, Centre of the Region Haná for Biotechnological and Agricultural Research, Faculty of Science, Palacký University and Institute of Experimental Botany ASCR, Olomouc, CZ-78371, Czech Republic; National University of Rosario, ARGENTINA

## Abstract

Phosphoglucose isomerase (PGI) catalyzes the reversible isomerization of glucose-6-phosphate and fructose-6-phosphate. It is involved in glycolysis and in the regeneration of glucose-6-P molecules in the oxidative pentose phosphate pathway (OPPP). In chloroplasts of illuminated mesophyll cells PGI also connects the Calvin-Benson cycle with the starch biosynthetic pathway. In this work we isolated *pgi1-3*, a mutant totally lacking pPGI activity as a consequence of aberrant intron splicing of the pPGI encoding gene, *PGI1*. Starch content in *pgi1-3* source leaves was ca. 10-15% of that of wild type (WT) leaves, which was similar to that of leaves of *pgi1-2*, a T-DNA insertion pPGI null mutant. Starch deficiency of *pgi1* leaves could be reverted by the introduction of a *sex1* null mutation impeding β-amylolytic starch breakdown. Although previous studies showed that starch granules of *pgi1-2* leaves are restricted to both bundle sheath cells adjacent to the mesophyll and stomata guard cells, microscopy analyses carried out in this work revealed the presence of starch granules in the chloroplasts of *pgi1-2* and *pgi1-3* mesophyll cells. RT-PCR analyses showed high expression levels of plastidic and extra-plastidic β-amylase encoding genes in *pgi1* leaves, which was accompanied by increased β-amylase activity. Both *pgi1-2* and *pgi1-3* mutants displayed slow growth and reduced photosynthetic capacity phenotypes even under continuous light conditions. Metabolic analyses revealed that the adenylate energy charge and the NAD(P)H/NAD(P) ratios in *pgi1* leaves were lower than those of WT leaves. These analyses also revealed that the content of plastidic 2-C-methyl-D-erythritol 4-phosphate (MEP)-pathway derived cytokinins (CKs) in *pgi1* leaves were exceedingly lower than in WT leaves. Noteworthy, exogenous application of CKs largely reverted the low starch content phenotype of *pgi1* leaves. The overall data show that pPGI is an important determinant of photosynthesis, energy status, growth and starch accumulation in mesophyll cells likely as a consequence of its involvement in the production of OPPP/glycolysis intermediates necessary for the synthesis of plastidic MEP-pathway derived hormones such as CKs.

## Introduction

Starch is a branched homopolysaccharide of α-1,4-linked glucose subunits with α-1,6-linked glucose at the branched points. Synthesized by starch synthases (SS) using ADP-glucose (ADPG) as the sugar donor molecule, this polyglucan accumulates as predominant storage carbohydrate in plants. Starch is found in the plastids of photosynthetic and non-photosynthetic tissues. Mature chloroplasts occurring in photosynthetically active cells possess the capacity of providing energy (ATP) and fixed carbon for the synthesis of starch during illumination. By contrast, production of long-term storage of starch taking place in amyloplasts of reserve organs such as tubers, roots and seed endosperms depends upon the incoming supply of carbon precursors and energy from the cytosol. This difference between the metabolic capacities of chloroplasts and amyloplasts has lead to the generally accepted view that the pathway(s) involved in starch production are different in photosynthetic and non-photosynthetic cells (for a review see [1]).

In leaves, up to 50% of the photosynthetically fixed carbon is retained within the chloroplasts during the day to synthesize starch [2,3], which is then remobilized during the subsequent night to support non-photosynthetic metabolism and growth by continued export of carbon to the rest of the plant. Due to the diurnal rise and fall cycle of its levels, foliar starch is termed “transitory starch”. Many environmental factors such as photoperiod, light quality, senescence, temperature, contact with microorganisms, etc., influence transitory starch metabolism [4–8]. Because starch is a major integrator in the regulation of plant growth to cope with fluctuations in the carbon and energy status of the plant [9] the synthesis of this polyglucan in leaves is highly regulated at multiple levels in response to light and sugar signals and hormones such as cytokinins (CKs) [10,11], abscisic acid [12,13] and brassinosteroids [14,15].

It is widely accepted that the whole photosynthesis-driven starch biosynthetic process occurring in mesophyll cells of leaves resides exclusively in the chloroplast [16–18]. According to this classical view of starch biosynthesis, starch is considered the end-product of a metabolic pathway that is linked to the Calvin-Benson cycle by means of the plastidic phosphoglucose isomerase (pPGI). This enzyme catalyzes the conversion of fructose-6-phosphate (F6P) from the Calvin-Benson cycle into glucose-6-phosphate (G6P), which is then converted into glucose-1-phosphate (G1P) by the plastidic phosphoglucomutase (pPGM). ADPG pyrophosphorylase (AGP) then converts G1P and ATP into inorganic pyrophosphate and ADPG necessary for starch biosynthesis. This view also implies that AGP is the sole source of ADPG, and functions as the major regulatory step in the starch biosynthetic process [17–20]. However, despite the monumental amount of data supporting the classic interpretation of transitory starch biosynthesis in mesophyll cells, mounting evidence previews the possible occurrence of important additional pathway(s) involving the cytosolic and plastidic compartments (reviewed in [1]).

In addition to its involvement in the connection of the Calvin-Benson cycle with the starch biosynthetic pathway in illuminated leaves, pPGI is involved in glycolysis and in the regeneration of G6P molecules in the oxidative pentose pathway (OPPP) in heterotrophic organs and non-illuminated leaves. pPGI is strongly inhibited by light [21] and by 3-phosphoglycerate (3PGA) [22], a Calvin-Benson cycle intermediate accumulating in the chloroplast during illumination that allosterically activates AGP [19,20]. Although these characteristics of pPGI, and the low stromal G6P/F6P ratio occurring in the illuminated chloroplast (far lower than the equilibrium constant for pPGI [22,23]) would indicate that this enzyme is inactive to some extent during illumination (and thus during transitory starch accumulation), genetic evidence showing that transitory starch biosynthesis occurs solely by the pPGI pathway has been obtained from characterization of starch-deficient mutants impaired in pPGI [24–28]. In *Arabidopsis*, such evidence has been obtained from the characterization of the *pgi1–1* and *pgi1–2* pPGI mutants accumulating up to 40% and 10% of the wild type (WT) starch content, respectively [27,28]. The *pgi1–1* allele has a single nucleotide substitution resulting in ca. 7% of the WT pPGI activity [26], whereas *pgi1–2* is a T-DNA insertion null mutant of *PGI1* that completely lacks pPGI activity [28].

*pgi1–2* leaves accumulate ca. 10-fold more starch than leaves impaired in pPGM and AGP [28–32], which would apparently conflict with the widely accepted idea that the whole photosynthesis-driven starch biosynthetic process solely involves the Calvin-Benson cycle-pPGI-pPGM-AGP-SS pathway in mesophyll cells. However, consistent with the idea that pPGI-pPGM-AGP is the sole starch biosynthetic pathway operating in mesophyll cells, Kunz et al. [28] showed that starch granules are restricted to both bundle sheath cells adjacent to the mesophyll and stomatal guard cells, and suggested that the occurrence of starch in these cells is due to the incorporation of cytosolic G6P into the chloroplast, where it is then metabolized into starch [28].

During our searches for starch deficient plants of Arabidopsis we isolated and characterized a mutant, designated as *pgi1–3*, totally lacking pPGI activity as a consequence of the aberrant splicing of intron 6 of the pPGI encoding gene, *PGI1*. We found that, similar to *pgi1–2* leaves, starch content in *pgi1–3* leaves was ca. 10–15% of that of WT leaves. Contrary to expectations, microscopy analyses carried out in this work revealed the presence of starch granules in the mesophyll cells of the two *pgi1* mutants. Subsequent biochemical characterization of *pgi1* plants showed that pPGI is an important determinant of photosynthesis, energy status, growth and starch accumulation in mesophyll cells likely as a consequence of its involvement in the production of OPPP/glycolysis intermediates necessary for the synthesis of plastidic 2-C-methyl-D-erythritol 4-phosphate (MEP)-pathway derived hormones such as CKs. The data also support the occurrence in mesophyll cells of important pPGI independent starch biosynthetic pathway(s) involving the cytosolic and chloroplastic compartments.

## Results

### Identification and molecular characterization of a new pPGI null allele

Data available from the Arabidopsis Information Resource (http://www.arabidopsis.org) on the pPGI encoding *At4g24620* gene (*PGI1*) includes the Cs92274 *PGI1* polymorfism occurring in ethyl methanesulfonate-mutagenized plants in the Landsberg erecta (L*er*) background. To identify its mutations site(s) we cultured plants from N92274 seeds obtained from the Nottingham Arabidopsis Stock Centre (NASC), and sequenced the *PGI1* gene.

**Fig 1:**
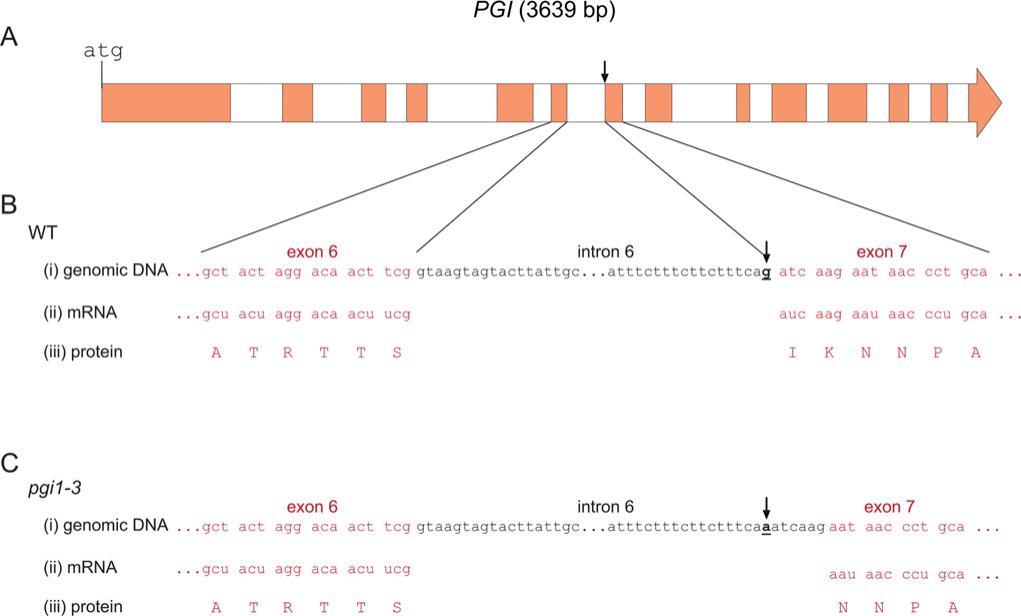
Molecular characterization of *pgi1–3*. (A) Genomic *PGI1* structure. In “B” and “C”, comparisons between the genomic DNA (i), the mRNA (ii), and the derived protein sequence (iii) of WT and the N92274 (*pgi1–3*) mutant. In “i” only the splicing sequences between the exon 6 and intron 6, and between the intron 6 and exon 7 are shown. Arrows indicate the point mutation site in *pgi1–3*. Exon sequences and their respective deduced amino acidic sequences are highlighted in red color.

*PGI1* contains 14 exons interrupted by 13 introns (Fig. 1A) [26]. Sequencing analyses revealed that the pPGI N92274 allele harbors a G to A transition in the UUCA**G**/AU sequence of the 3′splice donor site of intron 6 (Fig. 1B, C). To investigate whether this mutation would cause mis-splicing of the *PGI1* pre-mRNA in N92274, we amplified by reverse transcription (RT)-PCR the *PGI1* mRNA from both WT and the N92274 leaves. Sequencing of the resulting complete cDNAs revealed that the cDNA obtained from N92274 leaves lacks 6 nucleotides (ATCAAG) downstream the single point mutation site (S1 Fig.). The overall data thus showed that (i) the N92274 mutation leads to aberrant splicing of intron 6 during *PGI1* pre-mRNA maturation (Fig. 1C), resulting in the production of a 6 nucleotides shorter *PGI1* mRNA, (ii) the 3′ splicing site of intron 6 of N92274 *PGI1* pre-mRNA occurs in a non-canonical UCAAG/AA sequence located 6 nucleotides downstream the WT 3′splicing site of *PGI1* pre-mRNA, and (iii) N92274 *PGI1* encodes a protein that lacks two amino acids (Ile346-Lys347) occurring in the WT pPGI (Fig. 1C and S2 Fig.).

**Fig 2:**
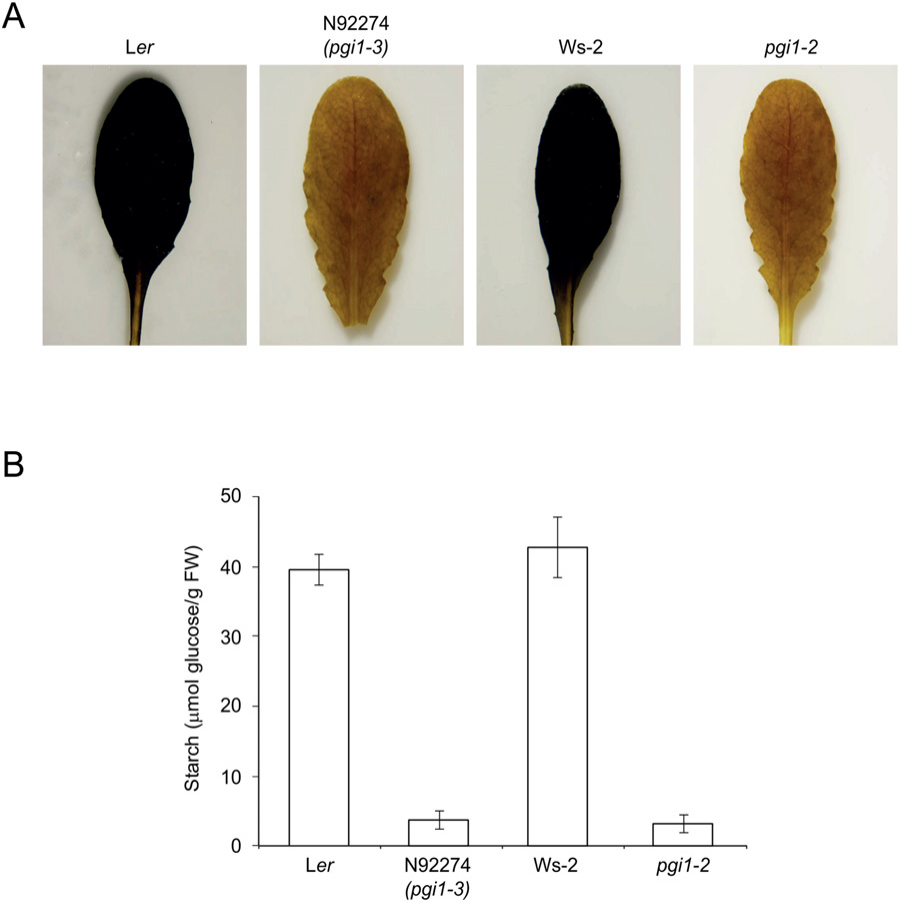
Leaves of the *pgi1–3* mutant accumulate low starch. (A) Iodine staining and (B) starch content of L*er*, *pgi1–3*, Ws-2 and *pgi1–2* leaves. Plants were cultured on soil under LD conditions and source leaves harvested from 30 DAG plants after 12 h of illumination. In “B” values represent the mean ± SE of determinations on five independent samples.

As a first step to investigate whether the above mutation affects pPGI activity we measured the starch content in leaves of N92274 plants and its corresponding WT (L*er*) plants. As reference, we also measured the starch content in the leaves of *pgi1–2* T-DNA insertion mutant totally impaired in pPGI activity [28] and its corresponding WT Wasilewskija (Ws-2) plants. Preliminary iodine staining analyses of leaves revealed that both N92274 and *pgi1–2* display a pale brown stain phenotype (Fig. 2A). This phenotype contrasts with the dark brown staining phenotype of WT leaves and the yellow stain phenotype of the near starch-less *aps1* and *pgm* leaves impaired in AGP and pPGM, respectively (S3 Fig.). This indicated the presence of reduced starch content in both N92274 and *pgi1–2* leaves. In line with this presumption, quantitative starch content measurement analyses revealed that starch content in both N92274 and *pgi1–2* leaves was ca. 10–15% of that accumulated by WT leaves (Fig. 2B).

**Fig 3:**
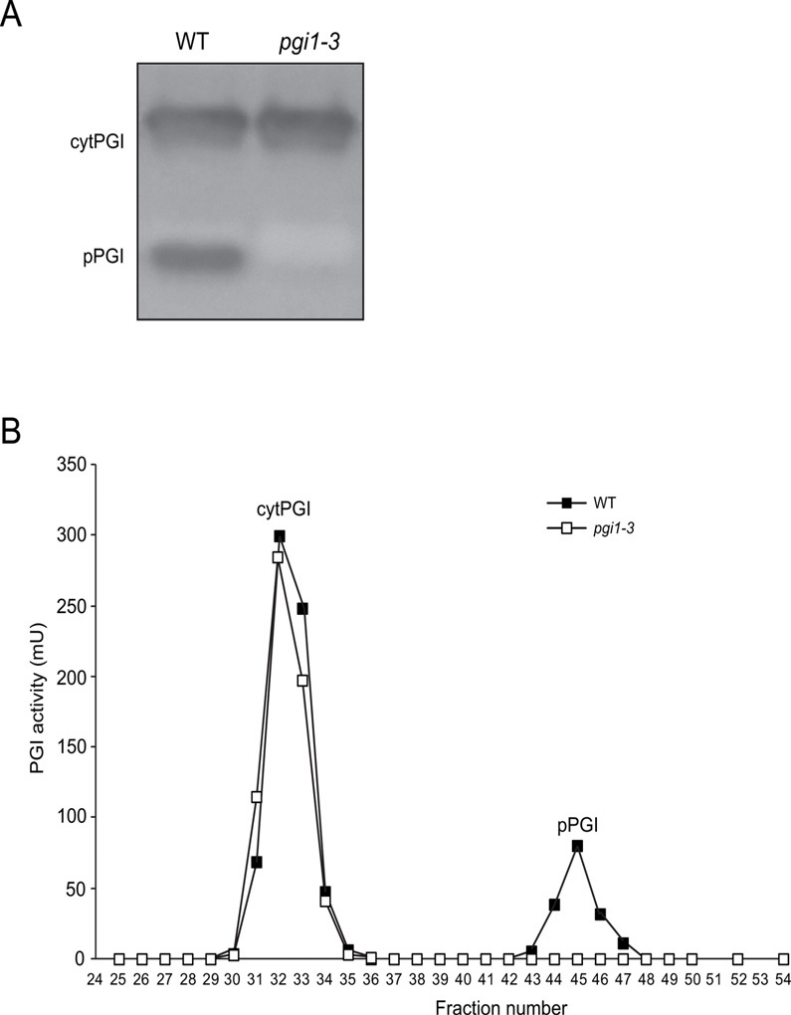
*pgi1–3* leaves lack pPGI activity. (A) PGI zymogram of proteins extracted from WT (L*er*) and *pgi1–3* leaves. (B) Q-sepharose chromatography profile of PGI activity in WT and *pgi1–3* leaves. In “B”, loaded WT extract contained 850 mU of total PGI activity, whereas *pgi1–3* extract loaded on the column contained 650 mU of PGI activity.

The above results indicated that the mutation in the pPGI N92274 allele (thereafter designated as *pgi1–3*) totally abolishes pPGI activity. To test this hypothesis we measured total PGI activity in *pgi1–3* plants. We also carried out zymogramic and Q-sepharose chromatographic analyses of PGI activity as described in Materials and Methods. Two PGI isozymes exist in Arabidopsis, one in the plastids and the other in the cytosol [26,33]. Typically, pPGI activity constitutes ca. 20–30% of the total cellular PGI [24,28]. Similarly to leaves of the *pgi1–2* T-DNA insertion mutant [28], total PGI activity in *pgi1–3* leaves (928 ± 52 mU/g FW) was ca. 80% of the WT PGI activity (1264 ± 134 mU/g FW). Zymogramic analyses of PGI activity revealed the occurrence of cytosolic PGI (cytPGI) and pPGI in WT leaves, but only cytPGI in *pgi1–3* leaves (Fig. 3A). PGI activity analyses of Q-sepharose chromatography eluted fractions revealed two activity peaks in WT leaves, whereas a single peak (corresponding to cytPGI) could be detected in *pgi1–3* leaves (Fig. 3B). The overall data thus provided strong evidence that the *pgi1–3* chloroplasts totally lack PGI activity.

**Fig 4:**
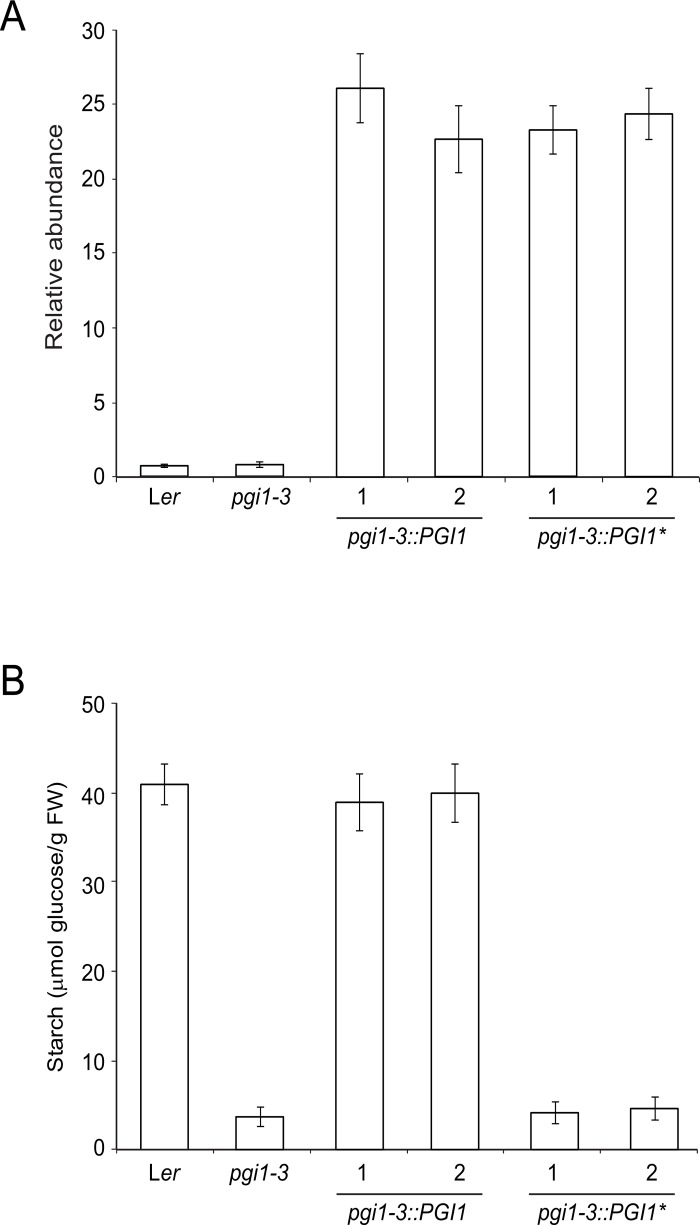
*pgi1–3* is a pPGI null allele. (A) RT-PCR of *PGI1* and (B) starch content in source leaves of WT (L*er*), *pgi1–3* and two independent lines each of *pgi1–3*::*PGI1* and *pgi1–3*::PGI1*. Plants were cultured on soil under LD conditions and leaves harvested from 30 DAG plants after 12 h of illumination. In “B” values represent the mean ± SE of determinations on five independent samples.

Whether *pgi1–3* is a pPGI null mutation was further investigated by generating and characterizing various *pgi1–3* plants expressing the *PGI1* encoding cDNA obtained from either WT or *pgi1–3* plants under the control of the CaMV 35S promoter (designated as *pgi1–3*::*PGI1* and *pgi1–3*::PGI1* plants, respectively). As shown in Fig. 4A, real time RT-PCR analyses showed that leaves of plants of two independent lines each of *pgi1–3*::*PGI1* and *pgi1–3*::PGI1* exhibited high expression levels of the transgene. Furthermore, leaves of plants of two independent *pgi1–3*::*PGI1* lines accumulated WT starch content whereas, similar to *pgi1–3* leaves, *pgi1–3*::PGI1* leaves accumulated ca. 10–15% of the WT starch content (Fig. 4B). The overall data thus further provided evidence that *pgi1–3* is a pPGI null allele.

### Mesophyll cells of *pgi1* null mutants contain starch

**Fig 5:**
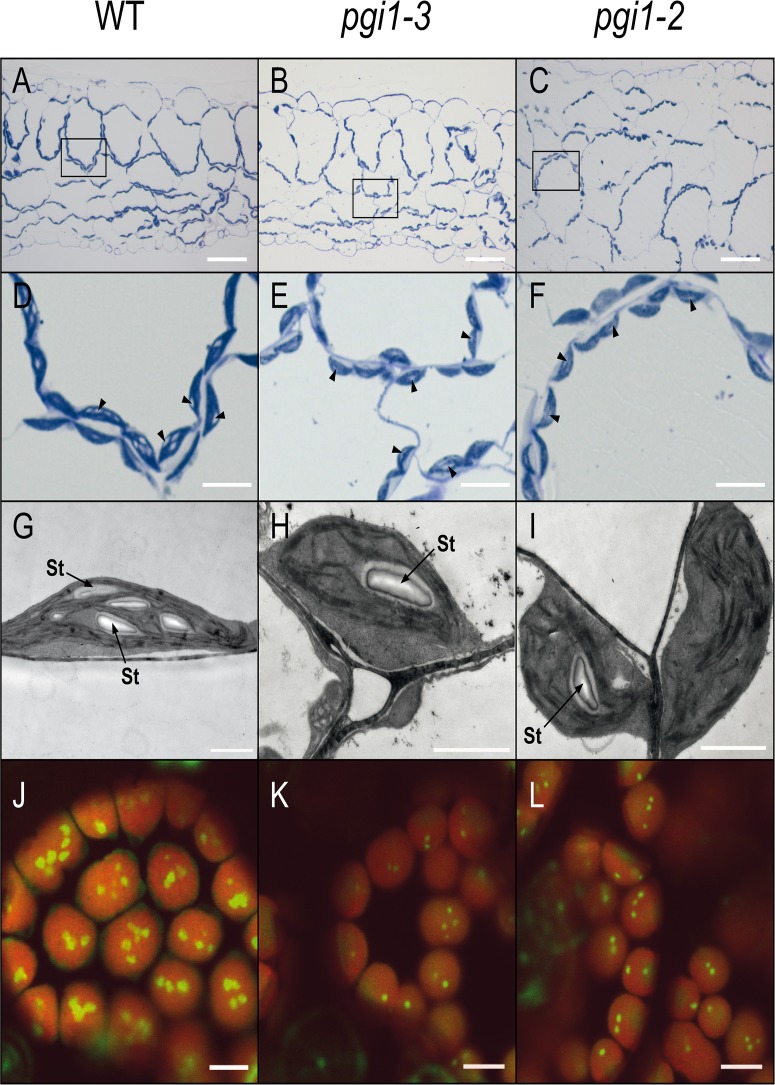
Microscopic analysis of starch granules in WT (L*er*), *pgi1–2* and *pgi1–3* source leaves. (A-C) Light microscopy of toluidine stained leaf sections (Bar = 50 μm). (D-F) Magnification of mesophyll sections indicated in A-C (Bar = 10 μm). (G-I) TEM of WT, *pgi1–2* and *pgi1–3* leaves. Bar = 2 μm. (J-L) CFM of leaves of GBSS-GFP expressing WT, *pgi1–2* and *pgi1–3* leaves. Bar = 5 μm. Plants were cultured on soil under LD conditions and source leaves harvested from 30 DAG plants after 12 h of illumination. In D-F, arrows indicate the position of starch granules. St: starch.

We carried out transmission electron microscopy (TEM) analyses of *pgi1–2* and *pgi1–3* mature leaves, and confocal fluorescence microscopy (CFM) analyses of mature leaves of granule bound starch synthase fused with green fluorescent protein (GBSS-GFP) expressing *pgi1–2* and *pgi1–3* plants. Preliminary light microscopy analyses of toluidine stained leaves showed that mesophyll cells of WT leaves produced several starch granules per chloroplast (Fig. 5A, D). Noteworthy, these analyses also revealed that chloroplasts of *pgi1–2* and *pgi1–3* mesophyll cells contain starch granules (Fig. 5B, C, E, F). These observations were further confirmed by TEM analyses of WT, *pgi1–2* and *pgi1–3* leaves (Fig. 5G-I), and CFM analyses of transgenic WT, *pgi1–2* and *pgi1–3* leaves expressing the starch granule marker GBSS-GFP (Fig. 5J-L).

**Fig 6:**
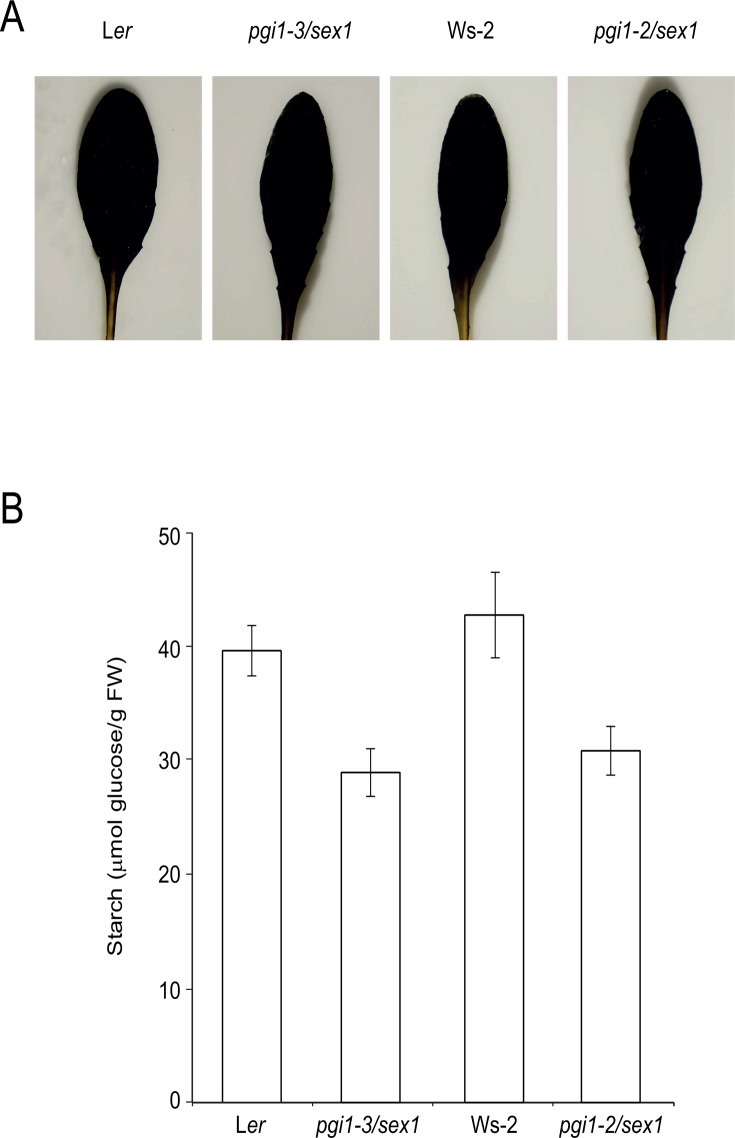
Introduction of *sex1* mutation into *pgi1* plants reverts the low starch content phenotype. (A) Iodine staining and (B) starch content of L*er*, *pgi1–3/sex1*, Ws-2 and *pgi1–2/sex1* leaves. Plants were cultured on soil under LD conditions and leaves harvested from 30 DAG plants after 12 h of illumination. In “B” values represent the mean ± SE of determinations on five independent samples.

SEX1 is required for β-amylase-mediated leaf starch mobilization during the night. Mutants impaired in this function accumulate high levels of starch in the mesophyll cells [34,35]. Whether mesophyll cells of *pgi1* null mutants accumulate starch was further investigated by characterizing *pgi1–2/sex1* and *pgi1–3/sex1* double mutants. The rationale behind this experimental approach was that, if *pgi1–2* and *pgi1–3* mesophyll cells do indeed accumulate starch, *pgi1–2/sex1* and *pgi1–3/sex1* leaves should accumulate more starch than *pgi1–2* and *pgi1–3* leaves, respectively. Confirming this presumption, both iodine staining (Fig. 6A) and starch content measurement analyses (Fig. 6B) revealed that *pgi1–2/sex1* and *pgi1–3/sex1* mature leaves accumulate exceedingly higher levels of starch than *pgi1–2* and *pgi1–3* leaves, respectively (see also Fig. 2).

### GPT2 is not involved in starch biosynthesis in mesophyll cells of *pgi1* leaves

**Fig 7:**
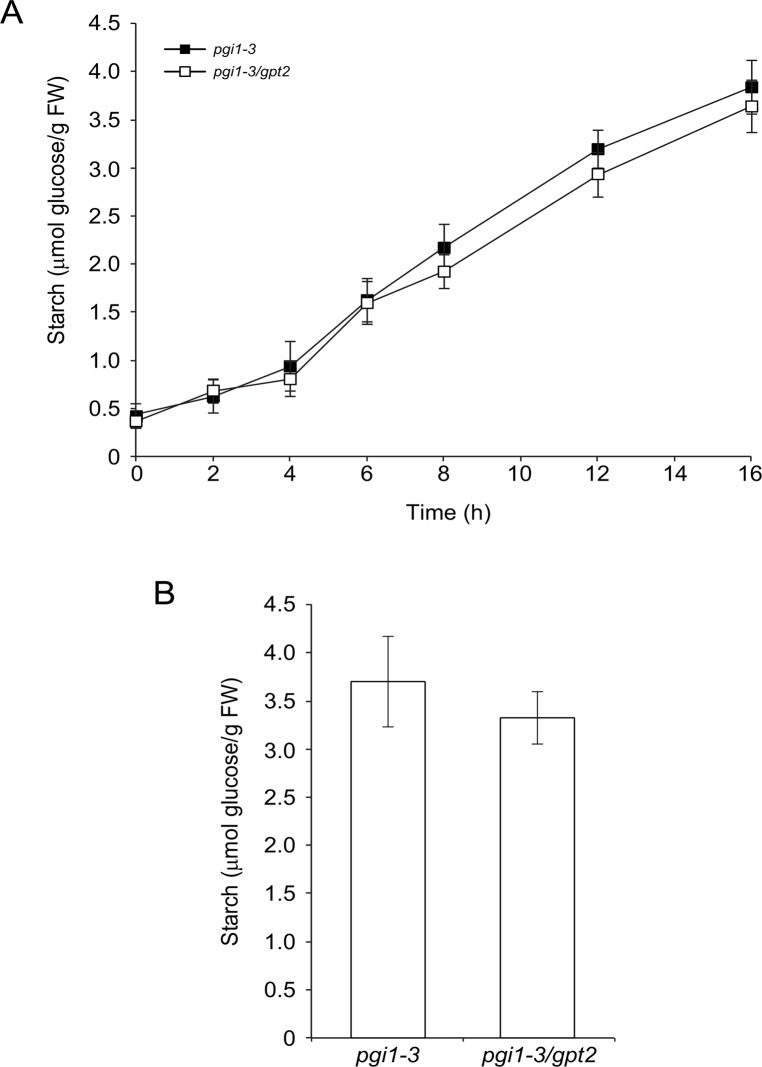
GPT2 is not involved in starch biosynthesis in mesophyll cells of *pgi1* leaves cultured on soil under LD and CL conditions. (A) Time-course of starch content in source leaves of *pgi1–3* and *pgi1–3/gpt2* plants cultured under LD conditions. Essentially the same results were obtained with *pgi1–2/gpt2* plants (not shown). (B) Starch content in source leaves of *pgi1–3* and *pgi1–3/gpt2* plants cultured under CL conditions. Leaves were harvested from 30 DAG plants. Values represent the mean ± SE of determinations on five independent samples. Each sample included leaves from 3 different rosettes.

Arabidopsis contains two functional plastidic G6P/Pi translocators (GPT) mainly expressed in heterotrophic tissues (GPT1 and GPT2) whose suggested role is delivery of G6P to non-green plastids as carbon skeletons for the synthesis of starch and fatty acids, or to drive the OPPP [36–41]. Kunz et al. [28] proposed that the occurrence of ca. 10% of the WT starch in *pgi1–2* leaves is ascribed to transport of G6P from the cytosol to the plastids of bundle sheath cells adjacent to the mesophyll and stomatal guard cells. The same authors reported that, unlike WT leaves, *pgi1–2* leaves exhibit substantial GPT activity as a consequence of the induction of *GPT2*, and proposed that GPT2 could partially contribute in the synthesis of starch in stomatal guard cells and bundle sheath cells of *pgi1–2* leaves [28]. Whether GPT2 is involved in the synthesis of starch in mesophyll cells of *pgi1* leaves was investigated by carrying out time-course analyses of the starch content in mature leaves of *pgi1* and *pgi1/gpt2* plants cultured under long day (LD) (16 h light/8 h dark) conditions. In these conditions *pgi1/gpt2* leaves accumulated as much starch as *pgi1* leaves (Fig. 7A). We also measured the starch content in leaves of *pgi1* and *pgi1/gpt2* plants cultured under continuous light (CL) conditions, and found that *pgi1* and *pgi1/gpt2* leaves accumulate comparable levels of starch (Fig. 7B). The overall data thus show that GPT2 plays a minor role in starch biosynthesis in mesophyll cells of mature *pgi1* leaves when plants are cultured under LD and CL conditions.

### Enzymatic characterization of *pgi1* leaves

**Fig 8:**
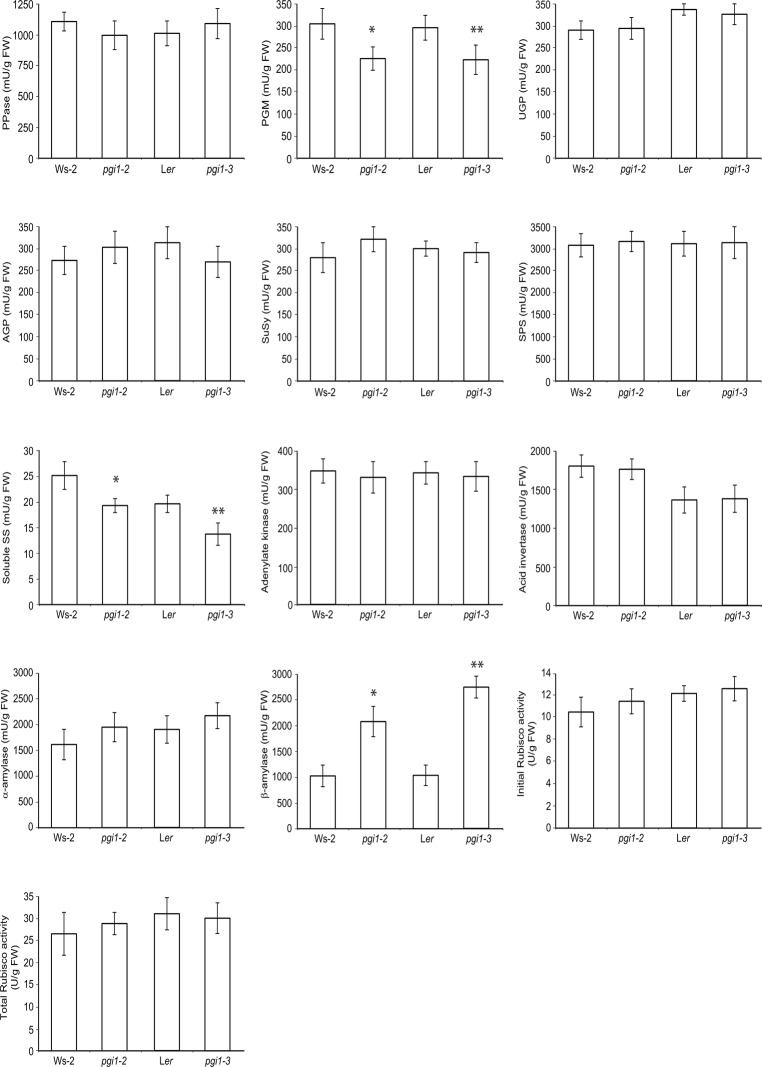
Activities of enzymes closely connected to starch and sucrose metabolism in source leaves of WT (L*er* and Ws-2), *pgi1–2* and *pgi1–3* plants. Plants were cultured on soil under LD conditions. Fully developed, source leaves were harvested from 30 DAG plants after 12 h of illumination. Values represent the mean ± SE of determinations on four independent samples. Asterisks indicate significant differences based on Student’s t-tests. (*P<0.05, *pgi1–2* vs. Ws-2; **P<0.05, *pgi1–3* vs. L*er*).

To examine for possible occurrence of pleiotropic effects that could determine the starch deficient phenotype of *pgi1* leaves we measured the maximum catalytic activities of a range of enzymes closely connected to starch and sucrose metabolism in mature leaves of *pgi1–2* and *pgi1–3* plants, and in leaves of their corresponding WT plants cultured under LD conditions. Only minor changes likely due to statistical variation were observed for AGP, alkaline pyrophosphatase (PPase), UDP-glucose (UDPG) pyrophosphorylase (UGP), sucrose-phosphate synthase (SPS), SuSy, acid invertase, α-amylase, adenylate kinase and ribulose 1,5-bisphosphate carboxylase oxygenase (Rubisco) in *pgi1* leaves (Fig. 8). However, both *pgi1–2* and *pgi1–3* leaves displayed low total PGM and soluble SS activities, and high β-amylase activity.

Increase of total β-amylase activity is a common feature of various mutants impaired in starch synthesis or breakdown [42]. Of the nine β-amylase-like proteins encoded in the *Arabidopsis* genome (BAM1–9), only BAM1–4 are plastidial and thus have direct access to starch [43–45]. BAM1 degrades starch during the day in both mesophyll and in guard cells subjected to heat shock and osmotic stress [7,46]. BAM3 is a major determinant of leaf starch degradation during the night [45], playing also an important role in starch degradation during the day upon cold shock [5]. BAM4 is a noncatalytic protein required for starch breakdown, acting upstream of BAM1–3 [45]. Increase of total β-amylase activity in mutants impaired in starch metabolism is largely due to enhanced extraplastidial β-amylase [42]. Consistently, RT-PCR analyses revealed that the expression levels of the extraplastidial BAM5 encoding gene in *pgi1* leaves are many fold higher than those of WT leaves (S4 Fig.). Noteworthy, these analyses also revealed that the expression levels of *BAM1–3* in *pgi1* leaves are exceedingly higher than those of WT leaves, the overall data suggesting that high plastidic β-amylase activity in *pgi1* leaves can be the consequence of high expression levels of both intra- and extra-plastidial β-amylases.

AGP activity is subjected to redox regulation of the small AGP subunit (APS1) [47,48]. To investigate whether the reduced levels of starch in the *pgi1* leaves could be ascribed to redox inactivation of APS1, we carried out APS1 immunoblot analyses of proteins from WT and *pgi1* leaves that had previously been extracted and electrophoretically separated under non-reducing conditions. In these conditions APS1 is present as a mixture of ca. 50 kDa active (reduced) monomers and ca. 100 kDa inactive (oxidized) dimers formed by intermolecular links involving Cys bridges. Consistent with previous reports [47,48], these analyses revealed that most of APS1 is largely oxidized (inactive) in both WT and *pgi1* leaves (S5 Fig.). These analyses also revealed that *pgi1* leaves accumulate identical amounts of ca. 50 kDa monomers and ca. 100 kDa dimers of APS1 than WT leaves, the overall data strongly indicating that the reduced starch content of *pgi1* leaves is not ascribed to redox inactivation of APS1.

### *pgi1* plants display a slow growth phenotype even under continuous light conditions

**Fig 9:**
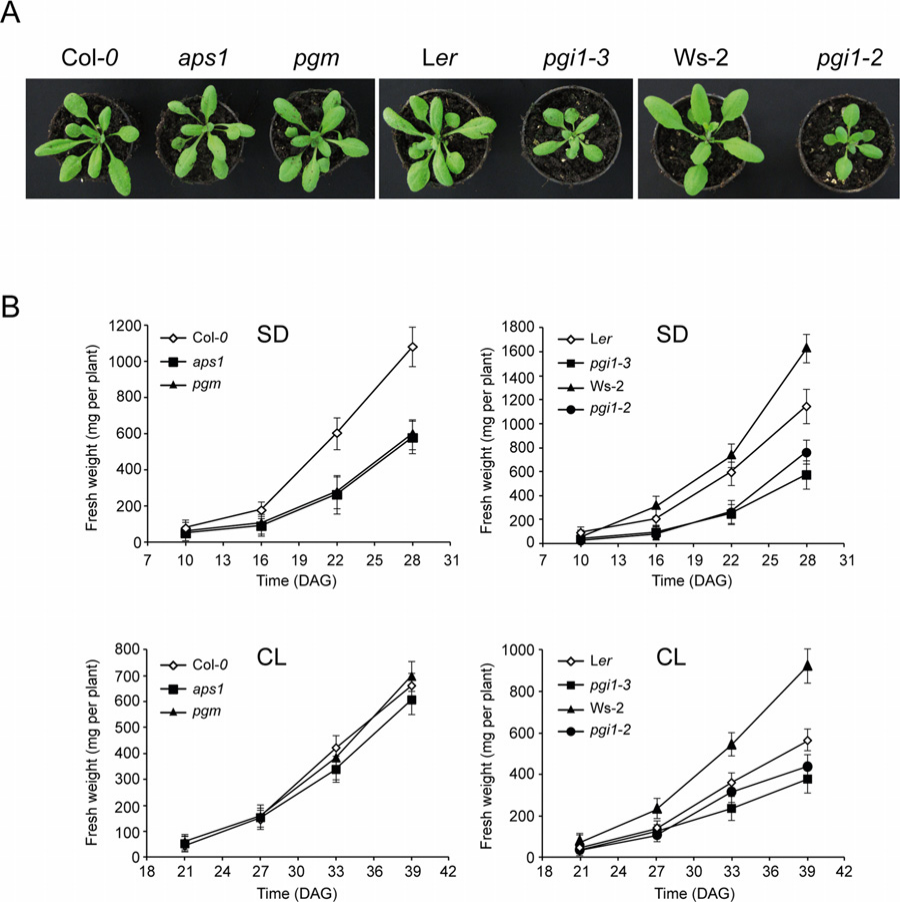
*pgi1–2* and *pgi1–3* leaves display a slow growth phenotype under SD and CL photoperiod conditions. (A) Photographs of 25 DAG WT, *aps1*, *pgm*, *pgi1–2* and *pgi1–3* plants cultivated in growth cabinets under CL conditions. (B) Time-course of FW of rosettes of WT, *aps1*, *pgm*, *pgi1–2* and *pgi1–3* plants cultured on soil under SD and CL conditions. In “B”, values represent the mean ± SE of determinations on four independent samples.

Transitory starch is a major determinant of plant growth [49]. The importance of starch turnover in plant growth is demonstrated by studies of mutants that are defective in starch synthesis and mobilization. Thus, near-starchless plants impaired in AGP or pPGM show a large inhibition of growth when cultured in short day (SD) conditions, but grow at the same rate as WT plants under CL photoperiod conditions [29,50–53]. Previous studies on *pgi1* mutants did not include analyses of plant growth under CL conditions [26,28,54]. We thus carried out time-course analyses of FW of rosettes of *pgi1–2*, *pgi1–3* and WT plants cultured either under SD (12 h light/12 h dark) or CL photoperiod conditions. We also carried out analyses of growth of near-starchless *aps1* and *pgm* mutants (Columbia (Col-*O*) background)) impaired in AGP and pPGM, respectively. As shown in Fig. 9, *aps1* and *pgm* plants displayed a marked slow growth phenotype when cultured under SD conditions, and grew as WT plants when cultured under CL conditions. In clear contrast, *pgi1–2* and *pgi1–3* displayed a slow growth phenotype at any photoperiod regime (Fig. 9) strongly indicating that (a) reduced starch turnover is not the reason of the slow growth phenotype of *pgi1* mutants, and (b) pPGI is an important determinant of plant growth.

### *pgi1* leaves have reduced photosynthetic capacity even under continuous light conditions

**Fig 10:**
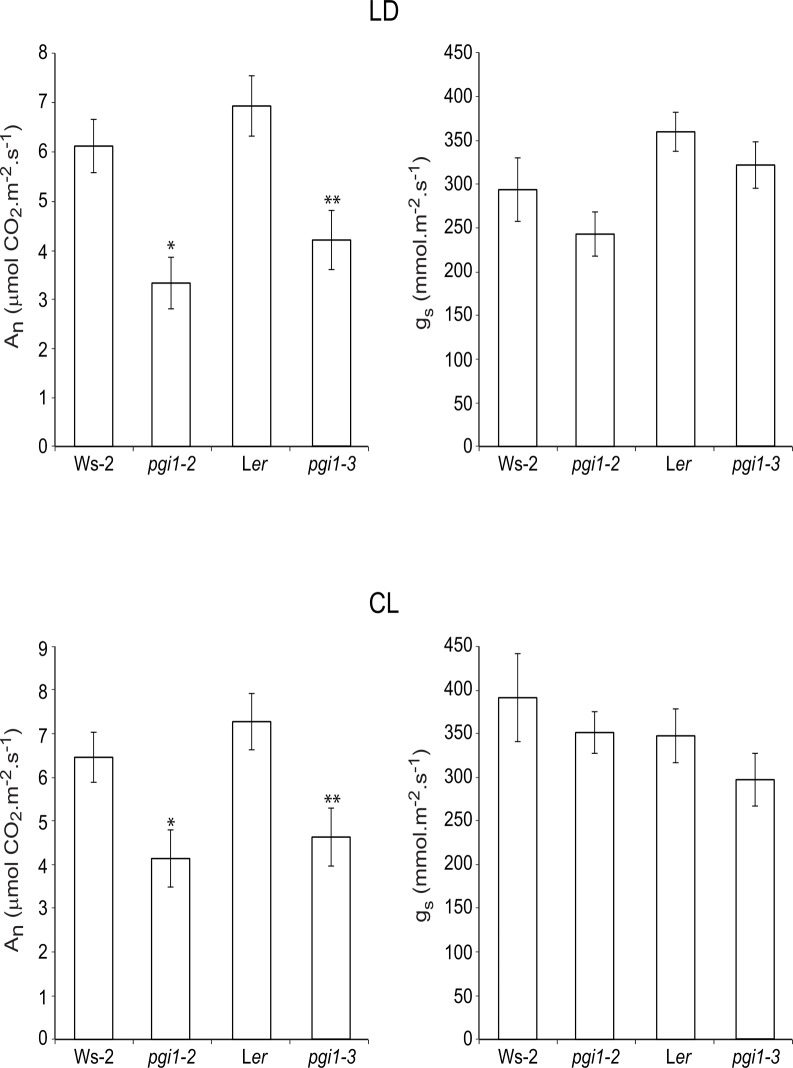
*pgi1* leaves have reduced photosynthetic capacity. The graphics represent net CO_2_ uptake (A) and stomatal conductance (*g*_*s*_) in source leaves of WT (Ws-2 and L*er*), *pgi1–2* and *pgi1–3* plants cultured on soil under SD and CL conditions. Values represent the mean ± SE of determinations on four independent samples. Asterisks indicate significant differences based on Student’s t-tests. (*P<0.05, *pgi1–2* vs. Ws-2; **P<0.05, *pgi1–3* vs. L*er*).

The low rates of growth and leaf starch accumulation of *pgi1* plants in both SD and CL conditions (see above) pointed to the possible occurrence of reduced photosynthetic capacity of *pgi1* leaves and/or altered mitochondrial respiration. We thus measured net photosynthetic CO_2_ fixation rates (A_n_) in mature leaves of *pgi1–2* and *pgi1–3* plants under saturating light intensity of 350 μmol m^-2^ s^-1^ and with a CO_2_ concentration of 450 μmol mol^-1^, and compared with those of WT plants when cultured under either LD or CL photoperiod conditions. We also analyzed the stomatal conductance (*g*_*s*_) under the same conditions. Moreover, we evaluated respiration rates in darkened *pgi1–2* and *pgi1–3* leaves. These analyses revealed that whereas respiration rates of darkened *pgi1* leaves were comparable to that of WT leaves (S6 Fig.), the photosynthetic capacities of *pgi1–2* and *pgi1–3* mature leaves were ca. 40–50% lower than that of WT leaves at any photoperiod regime. Furthermore, *g*_*s*_ values in *pgi1* plants were moderately (although not significantly) lower than those of WT plants (Fig. 10).

**Fig 11:**
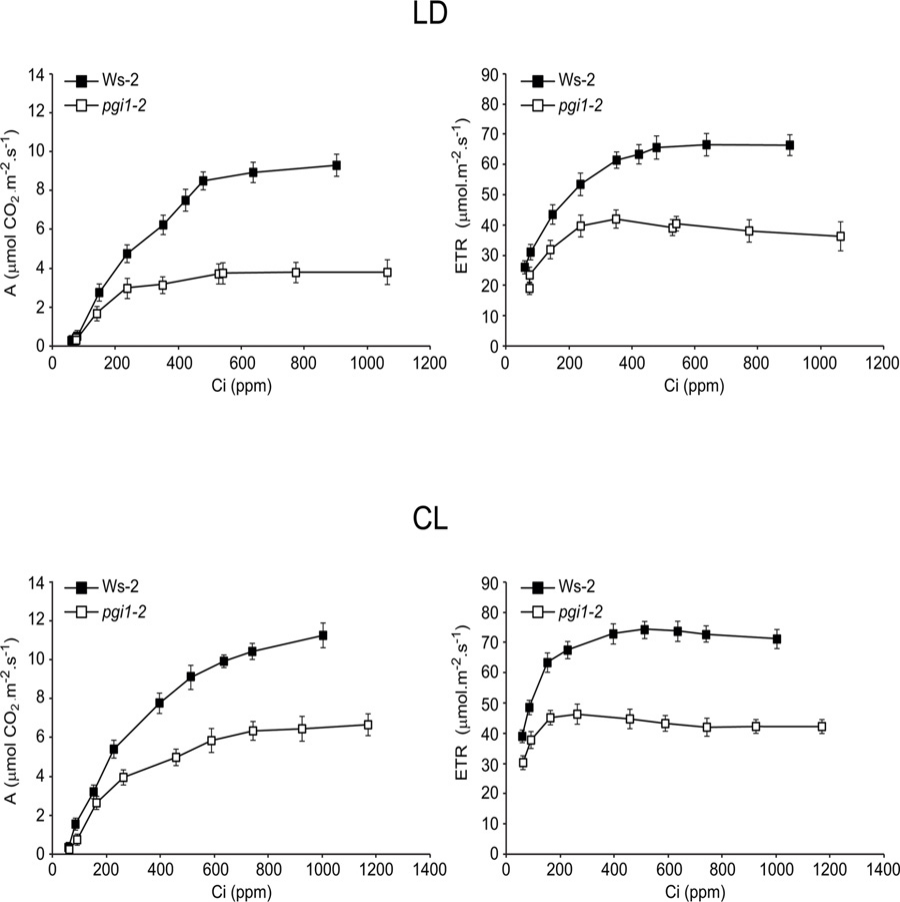
*pgi1–2* leaves have reduced photosynthetic capacity and ETR. (A) CO_2_ assimilation rates, and (B) photosynthetic electron transport at different intercellular CO_2_ concentrations in WT and *pgi1–2* source leaves. Plants were cultured on soil under LD conditions. Values represent the mean ± SE (n = 5).

A strong reduction in A_n_ in *pgi1* plants without a parallel strong reduction in *g*_*s*_ (and thus limitation in CO_2_ availability) would suggest the occurrence of biochemical limitations restricting photosynthesis. To test this hypothesis, we measured A_n_ in *pgi1* plants cultured under both LD and CL conditions, and under varying intercellular CO_2_ concentrations (Ci). We also measured the photosynthetic electron transport rate (ETR) with respect to varying Ci. As shown in Fig. 11, irrespective of the photoperiod conditions, *pgi1* plants had strongly reduced values of A_n_ when compared with WT plants. Analyses of the maximum carboxylation rate (Vcmax), the triose phosphate use (TPU) and the maximum rate of the electron transport (Jmax) (that is equivalent to the ribulose-1,5-bisP (RuBP) regeneration rate) calculated from the A_n_/Ci curves revealed that Vcmax of Rubisco in *pgi1* leaves is significantly lower than that of WT leaves (Table 1). This indicated that the reduction in A_n_ observed in *pgi1* plants was related to differences in *in planta* Rubisco activity. Furthermore, *pgi1* plants displayed significant reductions in Jmax and TPU with respect to WT plants, thus indicating a role of pPGI in the protection of the electron transport leading to the regeneration of RuBP and the capacity of the chloroplast reactions to use triose-phosphates. The absence of significant differences on Jmax/Vcmax between *pgi1* leaves and WT leaves indicate that reductions in Jmax and Vcmax in *pgi1* leaves are the consequence of similar factor(s), most likely perturbation in photosynthetic energy transduction in *pgi1* plants. Supporting this view, ETR values in WT plants were exceedingly higher than those of *pgi1* plants under any Ci conditions (Fig. 11).

**Table 1:** Photosynthetic parameters of WT (Ws-2 and L*er*), *pgi1–2* and *pgi1–3* source leaves. Plants were cultured under LD and CL conditions. Values represent the mean ± SE (n = 5).

Line	Jmax	Vcmax	TPU
Ws-2 (LD)	77.5 ± 6.6	56.0 ± 4.0	2.3 ± 0.29
*pgi1–2* (LD)	24.3 ± 1.2	21.6 ± 1.9	1.4 ± 0.20
Ws-2 (CL)	65.5 ± 3.4	29.4 ± 2.9	2.6 ± 0.21
*pgi1–2* (CL)	30.2 ± 1.9	17.7 ± 3.1	1.9 ± 0.15
L*er* (LD)	63.1 ± 6.5	38.9 ± 4.0	2.4 ± 0.22
*pgi1–3* (LD)	30.8 ± 6.5	15.1 ± 1.4	1.5 ± 0.12
L*er* (CL)	59.3 ± 4.4	64.5 ± 4.3	2.6 ± 0.30
*pgi1–3* (CL)	38.1 ± 2.7	36.7 ± 4.2	1.7 ± 0.19

### Metabolic characterization of *pgi1* leaves

**Fig 12:**
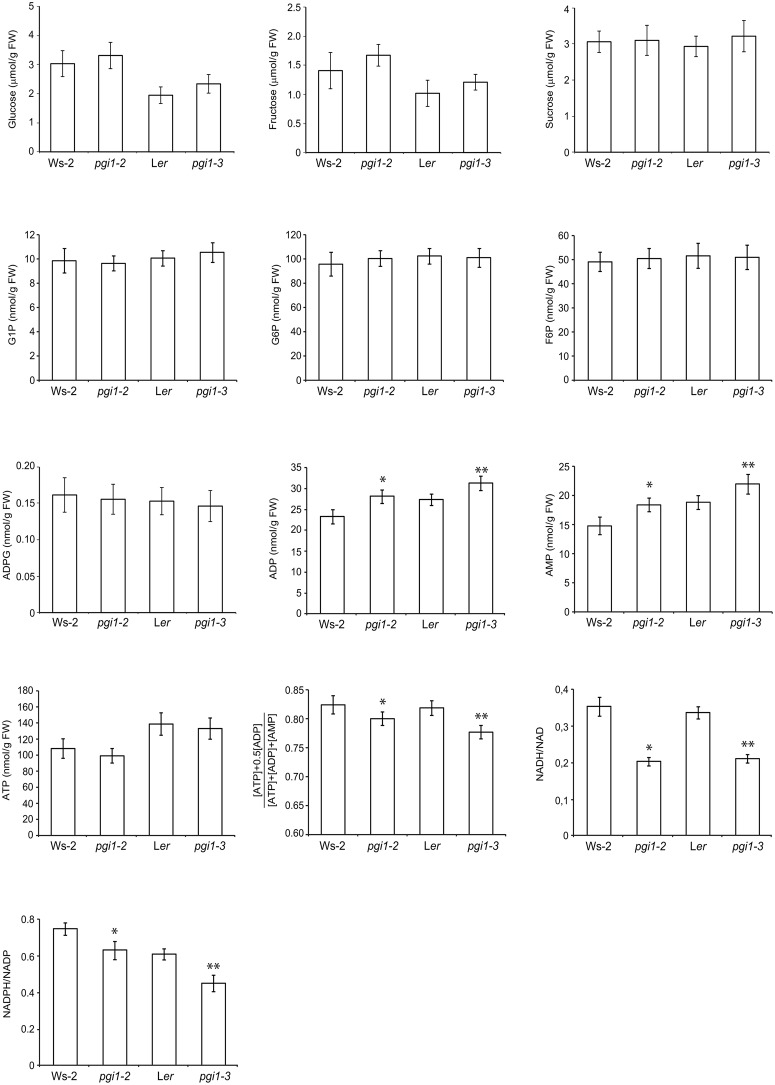
Metabolites content in mature leaves of WT (Ws-2 and L*er*), *pgi1–2* and *pgi1–3* plants cultured on soil under LD conditions. Fully developed, source leaves were harvested from 30 DAG plants after 12 h of illumination. Values represent the mean ± SE of determinations on five independent samples. Each sample included leaves from 3 different rosettes. Asterisks indicate significant differences based on Student’s t-tests. (*P<0.05, *pgi1–2* vs. Ws-2; **P<0.05, *pgi1–3* vs. L*er*).

We measured metabolites content in leaves of *pgi1–2*, *pgi1–3* and their corresponding WT plants cultured under LD conditions. Under these conditions *pgi1–2* and *pgi1–3* leaves accumulated nearly WT levels of glucose, fructose, sucrose, G6P, F6P and G1P (Fig. 12). Levels of ATP in *pgi1* leaves were slightly (but not significantly) lower than those of WT leaves, whereas levels of ADP and AMP in *pgi1–2* and *pgi1–3* leaves were significantly higher than in their corresponding WT leaves. Consistently, adenylate energy charge of *pgi1–2* and *pgi1–3* leaves was lower than that of WT leaves (Fig. 12). The NADPH/NADP and NADH/NAD ratios in *pgi1* leaves were significantly lower than in WT leaves (Fig. 12), which would indicate that pPGI is a determinant of the cellular redox potential. Consistent with previous reports showing the occurrence of important ADPG sources other than the pPGI-pPGM-AGP pathway [32,55], *pgi1–2* and *pgi1–3* leaves accumulated WT ADPG content (Fig. 12).

### *pgi1* leaves accumulate low levels of active forms of cytokinins derived from the plastidic MEP pathway

**Fig 13:**
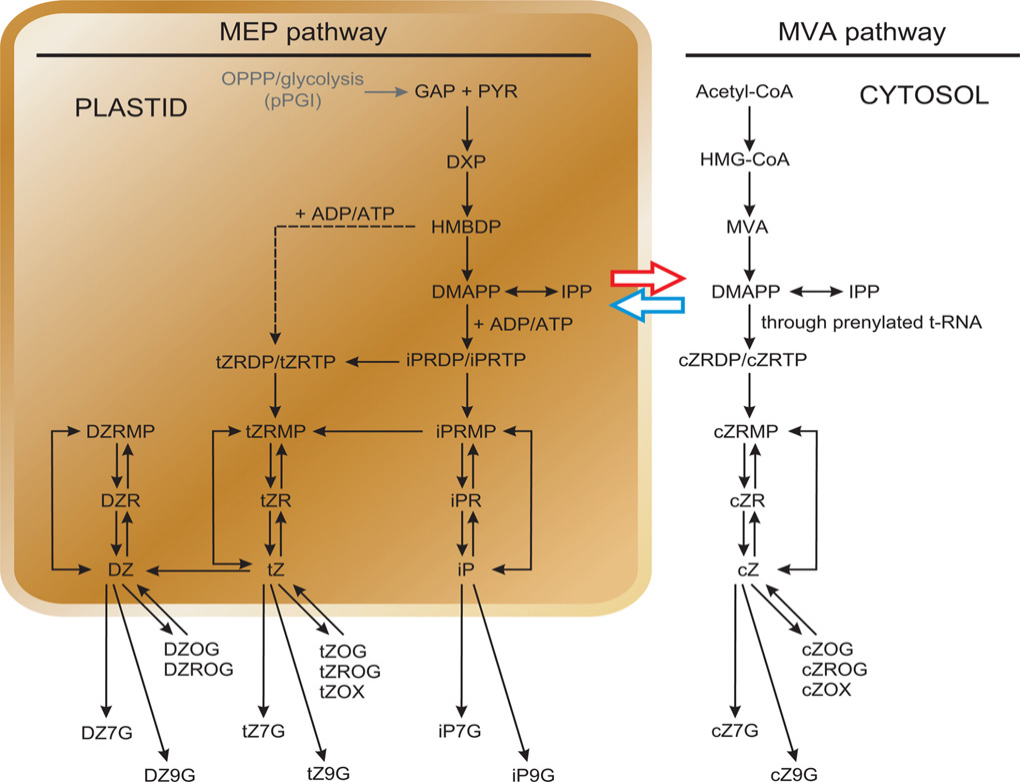
Scheme of CK biosynthesis through plastidic MEP- and cytosolic MVA-pathway. Black arrows show the biosynthesis, interconversions and metabolism flow of CKs in *Arabidopsis* cell (adapted from [121]). The dashed arrow indicates the iPRMP-independent pathway of tZ biosynthesis [104]. The blue and the red arrows indicate a hypothetical exchange of common precursor(s) between the MEP and MVA pathways (adapted from [101]). GAP, glyceraldehyde 3-phosphate; PYR, pyruvate; DXP, 1-deoxy-D-xylulose 5-phosphate.

pPGI is involved in the OPPP and glycolytic pathways in non-illuminated leaves and heterotrophic organs. Glyceraldehyde 3-phosphate (GAP) is a glycolytic and OPPP metabolic intermediate that acts as substrate for the initial reaction of the plastidic MEP pathway involved in the synthesis of isoprenoids [56–58] (Fig. 13). Therefore, pPGI could potentially act as a determinant for the synthesis of plastidic MEP-pathway derived isoprenoid compounds. Among different plastidic isoprenoid derived molecules, CKs have been shown to act as major determinants of growth, energy status, starch content and photosynthesis in mature leaves [10,11,59–63]. Therefore, we considered of interest to investigate the possible involvement of pPGI in CKs metabolism by measuring the levels of different CKs in mature leaves of both Ws-2 and *pgi1–2* plants. These analyses revealed that the lack of pPGI causes a decrease of the total content of plastidic-type, MEP pathway-derived isopentenyladenine (iP)- and *trans*-zeatin (tZ)-type CKs, mainly as a consequence of the reduction of the main precursors of the active CKs iPRMP and tZRMP (Table 2, Fig. 13). The tZRMP content in *pgi1–2* leaves was only 32% of that of WT leaves (Table 2). As a consequence, the levels of tZ (the most abundant biologically active CKs) and its riboside tZR in *pgi1–2* leaves were only 51% and 28% of those of WT leaves, respectively (Table 2). The iPRMP content in *pgi1–2* leaves was ca. 70% of that of WT leaves, whereas the iPR content in *pgi1–2* leaves was ca. 15% of that of WT leaves (Table 2), pointing to the possible occurrence of a general down-regulation of conversion of active CK free bases to their corresponding ribosides in *pgi1–2* plants. The content of the biologically less active DHZ in *pgi1–2* leaves was 50% of that of WT leaves (Table 2). The levels of the irreversibly glycosylated N9- and N7-glycosylated CKs (tZ9G, tZ7G, iP9G) and the reversibly *O-*glycosylated forms of tZ (tZOG and tZROG) were significantly lower than those of WT leaves (Table 2). Noteworthy, virtually no differences were found between the content of cytosolic mevalonate (MVA) pathway derived *cis-*zeatins (cZ) in *pgi1–2* and WT leaves (Table 2). The overall data would indicate that the lack of pPGI causes reduction of plastidic MEP pathway derived CKs, but not of cytosolic MVA pathway derived CKs in *pgi1–2* leaves.

**Table 2:** CKs content (pmol g^-1^ DW) in leaves of 30 DAG Ws-2 and *pgi1–2* plants. CK precursors, transport forms, active forms and glycosylated inactive forms are separated in two groups based on their origin from MEP and MVA pathway, respectively. Total sums and corresponding percentage is shown for individual forms. Symbols indicate significant differences according to ANOVA. *< 0.05; **<0.01; **<0.001; ns, not significant; n.a., not analyzed.

	MEP pathway (plastid) derived CKs			MVA pathway (cytosol) derived CKs		
		WS-2	*pgi1–2*		WS-2	*pgi1–2*
Precursors	iPRMP	465 ± 43.4	329 ± 34.5 ns	cZMRP	51.4 ± 8.5	48.2 ± 7.4 ns
	tZRMP	**845.6 ± 100.4**	269.1 ± 20.5 **			
	DHZMP	4.8 ± 0.3	3.5 ± 0.2 ns			
	∑ (%)	1315.4 (100%)	601.6 (45.7%)		(100%)	93.8%
Transport forms	iPR	**34.8 ± 4.4**	5.5 ± 0.5 ***	cZR	2.9 ± 0.2	2.0 ± 0.2 ns
	tZR	**100.2 ± 10.5**	28.4 ± 4.6 **			
	DHZR	**1.6 ± 0.2**	2.1 ± 0.1 *			
	∑ (%)	136.6 (100%)	36 (26.4%)		(100%)	69%
Active forms	iP	9.7 ± 0.8	11.8 ± 1.1 ns	cZ	1.8 ± 0.2	1.6 ± 0.1 ns
	tZ	**45.3 ± 4.2**	23.3 ± 1.5 **			
	DHZ	**0.8 ± 0.18**	0.4 ± 0.01 **			
	∑ (%)	55.8 (100%)	35.5 (63.6%)		(100%)	88.9%
Glycosylated (inactive) forms	iP7G	10.2 ± 0.7	12.7 ± 1.2 ns	cZ7G	n.a.	n.a.
	tZ7G	**65.9 ± 2.7**	42.5 ± 2.1 *			
	DHZ7G	6.1 ± 0.6	5.8 ± 0.1 ns			
	iP9G	**16.7 ± 2.0**	**7.9 ± 0.2** ***	cZ9G	**0.9 ± 0.1**	0.5 ± 0.0 **
	tZ9G	**445.4 ± 8.0**	216.7 ± 12.9 **			
	DHZ9G	4.2 ± 0.6	3.5 ± 0.2 ns			
	tZOG	193.4 ± 5.1	146.3 ± 8.9 ns	cZOG	**19.0 ± 2.1**	32.1 ± 0.9 **
	DHZOG	**1.8 ± 0.1**	3.9 ± 0.3 **			
	tZROG	18.5 ± 0.5	9.7 ± 1.3 ns	cZROG	23.0 ± 3.0	24.0 ± 2.5 ns
	DHZROG	1.2 ± 0.1	1.3 ± 0.2 ns			
	∑ (%)	763.4 (100%)	450.3 (59%)		42.9 (100%)	56.6 (131.9%)
Total ∑ (%)		2271.2 (100%)	1123.4 (49.5%)		47.6 (100%)	60.2 (126.5%)

### Exogenous application of CKs reverts the starch deficient phenotype of *pgi1* plants

**Fig 14:**
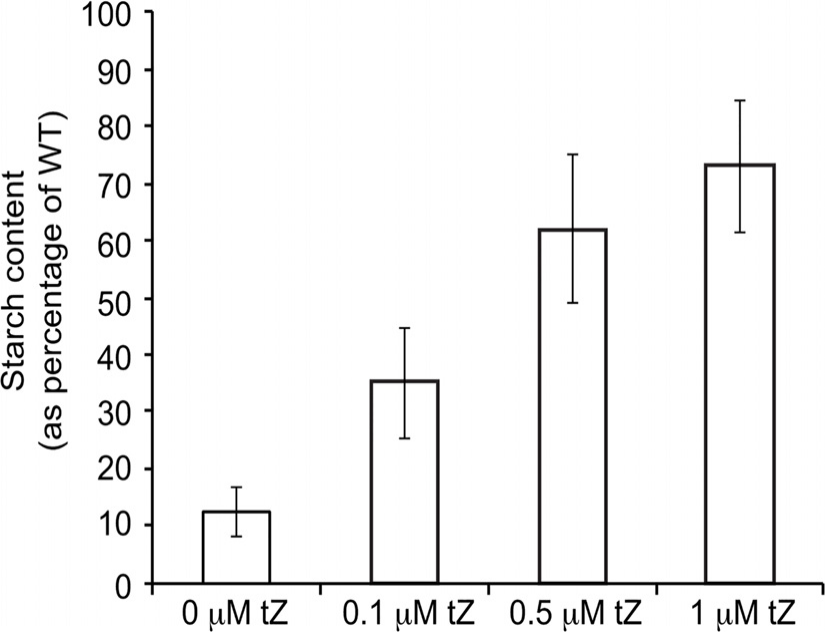
Starch content (as percentage of starch occurring in WT leaves) in leaves of *pgi1–2* cultured in MS medium including the indicated concentrations of tZ. Values represent the mean ± SE of determinations on four independent samples.

*pgi1* mutants exhibit symptoms indicative of reduced plastidic CKs content such as reduced size, low starch content and reduced photosynthetic capacity at any photoperiod condition (see above). Whether the low starch content phenotype of *pgi1* leaves could be the consequence, at least in part, of reduced plastidic CKs (see Table 2) was investigated by measuring the starch content in leaves of adult *pgi1* plants cultured for 2 days in solid MS medium supplemented with different concentrations of tZ. As shown in Fig. 14, these analyses revealed that exogenous CK application largely reverts the starch deficient phenotype of *pgi1* leaves.

## Discussion

The initial objective of this work was to characterize the *pgi1–3* mutant both at the molecular and biochemical levels, using *pgi1–2* as reference. Contrary to expectations, during the course of our studies we found that mesophyll cells of mature leaves of the two *pgi1* null mutants accumulate ca. 10–15% of the WT starch content (Figs. 2, 4 and 5). The discrepancy between our results showing the presence of starch granules in mesophyll cells of *pgi1* leaves and those of Kunz et al. [28] showing that starch granules are restricted to bundle sheath cells adjacent to the mesophyll and stomatal guard cells are likely ascribed to the use of different growth conditions. Thus, whereas Kunz et al. [28] carried out their experiments using plants cultured under SD conditions, we cultured the plants under LD and CL conditions. Another possible reason explaining the discrepancy between our results and those of Kunz et al. [28] is the use of different microscopic techniques.

The occurrence of starch granules in the chloroplasts of mesophyll cells conflicts with the widely accepted view that the whole photosynthesis-driven starch biosynthetic process in mesophyll cells solely occurs in the chloroplast by means of the Calvin-Benson cycle-pPGI-pPGM-AGP-SS pathway. Therefore, it is conceivable that at least 10–15% of the starch accumulated in the leaf mesophyll cells is produced by metabolic pathway(s) wherein (a) the Calvin-Benson cycle is not directly connected to the starch biosynthetic pPGM-AGP-SS pathway by means of pPGI, and (b) carbon units linked to starch biosynthesis are imported from the cytosol. Taking into account that (a) the photosynthetic capacity of *pgi1* leaves at any photoregime is exceedingly lower than that of WT plants (Figs. 10 and 11), and (b) the adenylate energy charge and cellular redox potential of *pgi1* leaves are lower than those of WT leaves (Fig. 12), it is highly likely that starch deficiency of *pgi1* leaves is partially the consequence of either reduced CO_2_ fixation capacity and/or low energy status and cellular redox potential, and not only the consequence of lack of pPGI-mediated flow between the Calvin-Benson cycle and the pPGM-AGP-SS starch biosynthetic pathway.

Chloroplasts of mature leaves are not capable of transporting G6P [64]. Consistently, studies of functional reconstitution of membrane proteins in proteoliposomes revealed that chloroplasts from Ws-2 and *Ler* plants do not transport G6P (cf. Table 2 in [28]). These studies also revealed that 97% of capacity of *pgi1–2* chloroplasts to transport G6P depends on GPT2, since G6P/Pi transport activity in proteoliposomes prepared from *pgi1–2/gpt2* leaves was only 3% of that found in proteoliposomes prepared from *pgi1–2* leaves (cf. Table 2 in [28]). Although this would indicate in principle that GPT2 could be involved in the incorporation of cytosolic G6P for its subsequent conversion into starch in *pgi1–2* mature leaves, results presented in Fig. 7 showing that the rate of starch accumulation in mature leaves of *pgi1* plants cultured under LD and CL conditions is comparable to that of leaves of *pgi1/gpt2* plants provide strong evidence that GPT2-mediated incorporation of cytosolic G6P into chloroplasts plays a minor role in the synthesis of starch in mesophyll cells of *pgi1* leaves. Although GPT1 only catalyzes the 3% of the total GP6/Pi transport activity in *pgi1–2* leaves, a possibility cannot be ruled out that GPT1 may contribute to some extent to the production of starch in mesophyll cells of *pgi1* plants cultured under LD and CL conditions.

Among a large group of plastidic metabolite transporters functionally related to the metabolism of sugars or sugar derivatives, none of them is able to transport G1P [37,65]. However, Fettke et al. [66] reported that the envelope membranes of chloroplasts of mesophyll cells possess a yet to be identified G1P transport machinery enabling the incorporation into the stroma of cytosolic G1P. According to these authors, however, such mechanism would only account for the accumulation of 1% of the WT starch, explaining the occurrence of trace amounts of starch in mutants impaired in pPGM. Thus, incorporation of cytosolic G1P into the chloroplast and its subsequent conversion into starch could minimally explain the accumulation of some starch in *pgi1* mesophyll cells chloroplasts. Chloroplasts possess a glucose transporter (pGlcT) [67] and hexokinase [68] potentially enabling the incorporation of cytosolic glucose and subsequent conversion into G6P thus bypassing the pPGI step in *pgi1* leaves. However *pglct* mutants accumulate WT levels of starch during the day [69], and GlcT has been shown to act in the export to the cytosol of glucose from the starch breakdown during the night rather than in the import of cytosolic glucose to the chloroplast [67,69]. Chloroplasts from mature leaves also possess a yet to be identified ADPG transport machinery [70]. Taking into account that a sizable pool of ADPG linked to starch biosynthesis has a cytosolic localization in leaves [32,55,71] it is likely that starch biosynthesis in mesophyll cells of *pgi1* mature leaves involves the cytosolic production of ADPG and its subsequent transport into the chloroplast and conversion into starch. Needless to say, further research will be necessary to identify the cytosolic hexose molecules entering the chloroplast for their subsequent metabolization into starch in *pgi1* leaves.

The occurrence of a starch biosynthetic pathway in mesophyll cells involving the incorporation into the chloroplast of cytosolic hexoses (either glucose, hexose-phosphates and/or ADPG) likely provides a clue to explain still enigmatic results reported almost 60 years ago using green leaves exposed to ^14^CO_2_ for a short period of time [72,73]. According to the widely accepted view of starch biosynthesis, leaves exposed to ^14^CO_2_ for a short period of time should produce starch with ^14^C symmetrically distributed in the glucose molecules. However, leaves shortly exposed to ^14^CO_2_ synthesized starch with ^14^C asymmetrically distributed in the glucose molecules [72,73]. Noteworthy, the same asymmetric distribution of ^14^C was found in the glucose moiety of hexose-phosphates, sucrose and nucleotide-sugars [72,73]. According to the models of starch biosynthesis involving the incorporation into the chloroplast of cytosolic hexoses (reviewed in [1]), triose-Ps exported from the chloroplast to the cytosol can be channeled into the OPPP, thereby leading to a randomization of the carbons that gives rise to the asymmetric ^14^C distribution observed in sucrose, hexose-phosphates and nucleotide-sugars (see Figure 4 in ref. [1]). Asymmetrically labeled cytosolic hexoses would enter the chloroplast, thus explaining why leaves shortly exposed to ^14^CO_2_ synthesize starch and sucrose with identical ^14^C asymmetric distribution. Also, the occurrence of metabolic pathways involving both the incorporation into the chloroplast of cytosolic hexoses, and the occurrence of simultaneous synthesis and breakdown of starch in the illuminated chloroplast (see [1] and references contained therein) likely provide clues to explain the rapid formation of radiolabelled maltose in leaves when plants are cultured in ^13^CO_2_ or ^14^CO_2_-enriched environments [74,75], and the asymmetric labeling of maltose formed by illuminated leaves cultured for a short period of time in ^14^CO_2_-enriched environment [74].

It is widely accepted that starch turnover is a major determinant of plant growth as demonstrated by studies of mutants that are defective in starch synthesis and mobilization. Some authors postulated that restricted growth of starch-deficient plants is the consequence of carbon starvation occurring every night due to the inability to accumulate starch during the day or to degrade it during the night [49,76,77]. Others postulated that acute deficiency of sugars occurring in starch mutants during the end part of the dark period temporary inhibits growth [78]. Restricted growth of starch-deficient plants has also been ascribed to regulatory imbalances in photosynthetic capacities and enzymatic activities triggered by high sugars during the day [29,79,80]. However, as shown in Fig. 12, *pgi1* leaves accumulate nearly WT levels of soluble sugars. Thus, factors other than high sugar levels must be responsible for the restricted growth (Fig. 9), reduced photosynthetic capacity (Figs. 10 and 11) and altered activities of some carbohydrate metabolism enzymes (Fig. 8) of *pgi1* mutants.

Results presented in Fig. 9 showing that *pgi1* plants cultured under CL conditions are smaller than WT plants, whereas the near-starchless *aps1* and *pgm* plants display a WT growth phenotype in the same culture conditions, provide strong evidence that (a) reduced starch turnover is not the reason of the slow growth phenotype of *pgi1* mutants, and (b) pPGI is an important determinant of plant growth. pPGI is involved in the regeneration of G6P molecules in the OPPP in heterotrophic organs and non-illuminated leaves. OPPP provides precursors for the synthesis of RNA, DNA and phenolic compounds such as aromatic amino acids, lignin, flavonoids and phytoalexins [81]. This metabolic pathway also provides NADPH necessary for biosynthetic redox reactions involved in lipid biosynthesis and nitrogen assimilation [36,40,82,83], and for NADP-thioredoxin reductase (NTRC) dependent processes such as supply of reductant necessary for detoxifying hydrogen peroxide in the dark, and maintaining the redox homeostasis of plastids which in turn determines plant growth and development [84–86]. Noteworthy, it has been reported that G6P metabolization within the OPPP is required for generating a signal that governs the regulation of root mediated acquisition of nitrogen and sulfur necessary for amino acid synthesis [87]. Also, previous reports have shown that, similar to plants impaired in pPGI, mutants impaired in other plastidic OPPP enzymes such as 6-phosphogluconolactonase display a reduced size phenotype [88,89]. Therefore, it is conceivable that the reduced size of *pgi1* mutants is ascribed, at least in part, to impairments in some OPPP-dependent processes occurring in heterotrophic organs that are important for growth.

Results presented in Figs. 10 and 11 show that the photosynthetic capacities of starch deficient *pgi1* plants are lower than that of WT plants at any photoregime. Noteworthy, recent studies have shown that the near-starchless *pgm1* mutant impaired in pPGM has WT photosynthetic CO_2_ fixation rates [90, our unpublished results], providing evidence that starch turnover exerts a minor influence on the photosynthetic capacity of the plant. This would strongly indicate that (a) the lack of pPGI, but not the reduced levels of starch, is the reason for the reduced photosynthetic capacity of *pgi1* plants, and (b) pPGI is an important determinant of the photosynthetic capacity of the plant. That *pgi1* mutants display reduced Vcmax, TPU and Jmax (Table 1) and ETR (Fig. 11) at any photoregime strongly indicates that impaired Rubisco carboxylation activity, together with limitations in RubP regeneration (as a consequence of reduced electron flux towards Rubisco carboxylation) and reduced capacity to use triose-phosphates are responsible for the low photosynthetic capacity of these mutants.

GAP and pyruvate are the substrates for the initial reaction of the plastidic MEP pathway involved in the synthesis of isoprenoid derived molecules such as CKs (Fig. 13) [58]. In non-illuminated leaves and heterotrophic organs GAP can be produced in the OPPP and glycolytic pathways involving pPGI. On the contrary, pyruvate biosynthesis in heterotrophic plastids largely depends on pPGI independent pathways, since enzymatic activities of the lower part of the glycolytic pathway such as phosphoglycerate mutase are marginally low in heterotrophic plastids [91–93]. Plastidic MEP pathway derived CKs are mainly synthesized in roots and transported to the aerial parts of the plant, where they regulate plant growth [94]. Noteworthy, in addition to their involvement in regulating plant growth and development, CKs act as major determinants of photosynthetic activity, Jmax and TPU by regulating the biogenesis of chloroplasts, and providing components of the electron transport chain, structural proteins and the enzymes for their formation [59–63,95]. Also, CKs maintain stomata open [96,97]. Moreover, CKs exert a positive effect on starch accumulation both in leaves and heterotrophic sink organs [10,11,98], most likely by regulating the expression of starch metabolism related genes [99]. Importantly, results presented in Table 2 showed that total levels of plastidic MEP pathway derived forms of CKs in *pgi1–2* leaves are low when compared with WT leaves, which provides evidence that pPGI is an important determinant of plastidic MEP pathway derived CKs (see below). Therefore, it is conceivable that the low net CO_2_ assimilation rate, Jmax, TPU and ETR (Figs. 10 and 11) (Table 1) as well as the reduced growth, energy and redox potential, and leaf starch content phenotypes of *pgi1* plants (Figs. 2, 5, 9 and 12) can be ascribed, at least in part, to the low content of active forms of plastidic MEP pathway derived CKs. In this respect we must emphasize that, similar to *pgi1* plants, CKs-deficient plants have reduced size and accumulate low starch in their source leaves [10,60,100]. That exogenous application of CKs partially reverted the reduced starch content phenotype of *pgi1* leaves (Fig. 14) further supports the view that the low starch content phenotype of *pgi1* leaves can be partially the consequence of reduced pPGI-mediated production of CKs.

In Arabidopsis the prenyl group of tZ- and iP-type CKs is mainly produced through the plastidic MEP pathway, whereas a large fraction of the prenyl group of cZ derivatives is provided by the cytosolic MVA pathway [101]. In *pgi1* leaves the most dramatic decrease in CKs content was found in the levels of CKs derived from the plastidic MEP pathway (iP- and tZ-type CKs), but not in the levels of cytosolic MVA-pathway dependent cZ-type CKs (Table 2). This would strongly indicate that pPGI is an important determinant of biosynthesis of CKs in plastids, but not in cytosol. Visible differences between WT and *pgi1–2* leaves were found in the levels of iP- and tZ-type CKs (Table 2). While the level of iPRMP (the product of the first dimethylallyl diphosphate (DMAPP)-dependent step of CK biosynthesis) in *pgi1–2* leaves was 70% of that occurring in WT leaves, the level of tZRMP in *pgi1–2* leaves was only 32% of that occurring in WT leaves (Table 2). Noteworthy, while the lack of pPGI in *pgi1–2* leaves resulted in a 50% reduction of the level of tZ free base, the lack of pPGI did not affect the level of iP free base at all (Table 2). The relatively high level of iPRMP and iP in *pgi1–2* leaves could be explained by the transport of DMAPP from cytosolic MVA pathway into plastids [101] to increase the DMAPP pool accessible for plastid localized isopentenyltransferases (IPTs). Such mechanism of common isoprenoid precursors (e.g. isopentenyl diphosphate or DMAPP) exchange between the cytosol and plastids was proposed to explain MVA-derived contribution to plastidic biosynthesis of gibberellins [102]. Another explanation for the relatively high level of iPRMP and iP in *pgi1–2* leaves can be that MVA-derived DMAPP serves as a substrate for AtIPT4, the cytosolic IPT isoform capable of *de novo* biosynthesis of iP, which can increase the overall iP cell pool [101]. It is worth mentioning here that tZRMP can be formed by the iPRMP-dependent pathway through hydroxylation of its side chain by CYP735A [103], and also directly through the iPRMP-independent pathway, utilizing a yet unknown side-chain donor of terpenoid origin [104] (Fig. 13). In this last respect, it can be speculated that the side-chain precursor is 1-hydroxy-2-methyl-2-butenyl 4-diphosphate (HMBDP), which is downstream of GAP in the MAP pathway (Fig. 13). The dramatic decrease of tZRMP and tZ levels can be thus explained as a combination of the lack of DMAPP needed to form iPRMP that is subsequently hydroxylated to tZRMP, together with the lack of a prenyl side chain precursor to form tZRMP.

Results presented in this work have provided evidence that pPGI is an important determinant of plastidic MEP pathway derived CKs likely as a consequence of its requirement in the production of GAP. GAP is the substrate for the initial reaction of the plastidic MEP pathway involved in the synthesis of isoprenoid derived molecules other than CKS such as gibberelins, abscisic acid, strigolactones, brassinosteroids, monoterpenes, carotenoids, tocopherols and prenylquinones (Fig. 13), some of them mutually interacting and acting as important determinants of growth, photosynthetic capacity, starch content and energy status of the plant [14,15,56–58]. It is thus likely that the reduced size and low starch content phenotypes of *pgi1* mutants are largely the consequence of changes in the overall plastidic MEP pathway-derived isoprenoid metabolism and its regulated processes. Needless to say, further research will be necessary to test this hypothesis.

## Materials and Methods

### Plants, growth conditions and sampling

The work was carried out using *Arabidopsis thaliana* L. (Heynh) (ecotypes L*er*, Col-*O* and Ws-2), the NASC N92274 (*pgi1–3*), the *pgi1–2* mutant [28], the *aps1*::*T-DNA* mutant (SALK_040155) [31], the *pgm*::*T-DNA* mutant (GABI_094D07), the *gpt2*::*T-DNA* mutant (GABI_454H06), *pgi1–3* plants expressing either *PGI1* or PGI1*, the *pgi1–3/gpt2* and *pgi1–2/gpt2* double mutants and the *pgi1–2/sex1* and *pgi1–3/sex1* double mutants. The *pgi1–2/sex1* and *pgi1–2/gpt2* double mutants were confirmed by PCR using the oligonucleotide primers listed in S1 Table. The *35S-PGI1* and 35S-PGI1* plasmid constructs utilized to produce *PGI1* or PGI1* expressing *pgi1–3* plants were produced as illustrated in S7 Fig. *pgi1–3* plants expressing GBSS-GFP were produced using the *35S-GBSS-GFP* plasmid construct [105] whereas *pgi1–2* plants expressing GBSS-GFP were produced using the 35S-GBSS-GFP* plasmid construct produced as illustrated in S8 Fig. The plasmid constructs were transferred to *Agrobacterium tumefaciens* EHA105 cells by electroporation and utilized to transform *Arabidopsis* plants according to [106]. Transgenic plants were selected on the appropriate antibiotic-containing selection medium.

Unless otherwise indicated plants were cultured in soil in growth chambers under the indicated photoperiodic conditions (light intensity of 90 μmol photons sec^-1^ m^-2^) and at a constant temperature of 22°C. Harvested source leaves were immediately freeze-clamped and ground to a fine powder in liquid nitrogen with a pestle and mortar.

To analyze the effects of exogenously applied CKs on starch content plants were grown *in vitro* on MS agar plates at a constant temperature of 22°C under LD conditions. Three-weeks old plants were then transferred to MS agar plates containing the indicated concentrations of tZ. After two additional days leaves were harvested, and starch content was measured as described below.

### Enzyme assays

One g of the frozen powder (see above) was resuspended at 4°C in 3 ml of 100 mM HEPES (pH 7.5), 2 mM EDTA and 2 mM dithiothreitol, 1 mM PMSF and 10 ml/L protease inhibitor cocktail (Sigma P9599), and centrifuged at 14,000 x g for 20 min. The supernatant was desalted by ultrafiltration on Vivaspin 500 centrifugal concentrator (Sartorius) and the protein extract thus obtained was assayed for enzymatic activities. AGP and UGP activities were measured following the two-step assay method described in [48]. PGI and SuSy were measured as described in [24] and [107], respectively. Adenylate kinase was assayed in the two directions of the reaction as described in [108] using an HPLC system (Waters corporation) fitted with a Partisil 10-SAX column. PGM and acid invertase were assayed as described in [29] and [109], respectively. Rubisco activity was measured according to [110]. Amylolytic activities were assayed as described in [111]. PPase and SPS were measured as described in [74]. SS activity was measured in two steps: (1) SS reaction and (2) measurement of ADP produced during the reaction. The SS assay mixture contained 50 mM HEPES (pH 7.5), 6 mM MgCl_2_, 3 mM dithiothreitol, 1 mM ADPG and 3% glycogen. After 5 min at 37°C reactions were stopped by boiling the assay mixture for 2 min. ADP was measured by HPLC on a Waters Associate’s system fitted with a Partisil-10-SAX column. One unit (U) is defined as the amount of enzyme that catalyzes the production of 1 μmol of product per min.

### Non-reducing western blot analyses of AGP

For non-reducing western blots of AGP, 50 mg of the homogenized frozen material (see above) was extracted in cold 16% (w/v) TCA in diethyl ether, mixed, and stored at -20°C for at least 2 h as described in [48]. The pellet was collected by centrifugation at 10,000 x g for 5 min at 4°C, washed 3 times with ice-cold acetone, dried briefly under vacuum, and resuspended in 1x Laemmli sample buffer containing no reductant. Protein samples were separated on 10% SDS-PAGE, transferred to nitrocellulose filters, and immunodecorated by using antisera raised against maize AGP as primary antibody [48], and a goat anti-rabbit IgG alkaline phosphatase conjugate (Sigma) as secondary antibody.

### Chromatographic separation of cytPGI and pPGI

Chromatographic separation of the two PGI isoforms was conducted using an AKTA FPLC from Amersham Pharmacia Biotech. Protein extracts of WT and *pgi1–3* leaves (see above) were loaded onto a HiLoad 16/10 Q-sepharose HP anion exchange column (Amersham Pharmacia Biotech) equilibrated with 50 mM HEPES (pH 7.5). After washing the column, the adsorbed proteins were eluted with a linear 0–0.8 M NaCl gradient in 50 mM HEPES (pH 7.5). The flow rate was 5 ml/min and 2.5 ml fractions were collected. Fractions were analyzed for PGI activity as described above.

### Native gel assay for PGI activity

PGI zymograms were performed as described in [29]. Protein extracts (see above) of both WT and *pgi1* leaves were loaded onto a 7.5% (w/v) polyacrylamide gel. After electroforesis gels were stained by incubating in darkness at room temperature with 0.1 M Tris-HCl (pH 8.0), 5 mM F6P, 1 mM NAD^+^, 4 mM MgCl_2_, 0.2 mM methylthiazolyldiphenyl-tetrazolium bromide (Sigma M5655) and 0.25 mM phenazine methosulfate (Sigma P9625) and 1 U/mL of G6P dehydrogenase from *Leuconostoc mesenteroides* (Sigma G8404).

### Analytical procedures

For determination of metabolites content, fully expanded source leaves of 30 days after germination (DAG) plants were harvested at the indicated illumination period, freeze-clamped and ground to a fine powder in liquid nitrogen with a pestle and mortar. ADPG content was measured by HPLC-MS/MS as described in [55]. For measurement of sucrose, glucose and fructose, a 0.1 g aliquot of the frozen powder was resuspended in 1 mL of 90% ethanol, left at 70°C for 90 min and centrifuged at 13,000 x g for 10 min. For measurement of G6P, F6P and G1P 0.5 g aliquot of the frozen powdered tissue was resuspended in 0.4 ml of 1 M HClO_4_, left at 4°C for 2 h and centrifuged at 10,000 x g for 5 min. The supernatant was neutralized with K_2_CO_3_ and centrifuged at 10,000 x g. Sucrose, glucose, fructose, F6P, G6P and G1P from supernatants were determined by HPLC with pulsed amperometric detection on a DX-500 Dionex system. NADP(H) and NAD(H) were measured as described in [112]. Starch was measured by using an amyloglucosydase—based test kit (Boehringer Mannheim, Germany). For measurement of adenine nucleotides a 0.5 g aliquot of the frozen powder was resuspended in 0.4 ml HClO_4_, left at 4°C for 2 h and centrifuged at 10,000 x g for 5 min. The supernatant was neutralized with K_2_CO_3_ and centrifuged at 10,000 x g. Nucleotides content in the supernatant was measured by HPLC (Waters corporation) fitted with a Partisil 10-SAX column as described in [113]. Recovery experiments were carried out by the addition of known amounts of metabolites standards to the frozen tissue slurry immediately after addition of extraction solutions.

For determination of CKs levels, aliquots of the frozen leaves (see above) were lyophilized and CKs were quantified according to the method described in [114].

### Iodine staining

Leaves harvested at the end of the light period were fixed by immersion into 3.7% formaldehyde in phosphate buffer. Leaf pigments were then removed in 96% ethanol. Re-hydrated samples were stained in iodine solution (KI 2% (w/v) I_2_ 1% (w/v)) for 30 min, rinsed briefly in deionized water and photographed.

### Gas exchange determinations

Fully expanded apical leaves were enclosed in a LI-COR 6400 gas exchange portable photosynthesis system (LI-COR, Lincoln, Nebraska, USA). The gas exchange determinations were conducted at 25°C with a photosynthetic photon flux density of 350 μmol m^-2^ s^-1^. A_n_ was calculated using equations developed by [115]. *g*_*s*_ values were determined as described in [116]. From the A/Ci curves, Vcmax, TPU and Jmax were calculated according to [117]. To avoid miscalculation of A_n_ and Ci due to leakage into the gasket of the gas analyzer, we performed CO_2_ response curves using an empty chamber. The values obtained for A_n_ and Ci in the empty chamber were compared with those of the chamber filled with a leaf and substracted from the values obtained with the empty chamber. ETR values were calculated according to [118] as Φ_PSII_ x PPFD x 0.84 x 0.5, where PPDF is the photosynthetic photon flux density incident on the leaf, 0.5 was used as the fraction of excitation energy distributed to PSII [119] and 0.84 as the fractional light absorbance [120]. The rate of mitochondrial respiration in the dark was determined by measuring the rate of CO_2_ evolution in the dark.

### Real-time quantitative PCR

Total RNA was extracted from leaves using the trizol method according to the manufacturer’s procedure (Invitrogen). RNA was treated with RNAase free DNAase (Takara). 1.5 μg RNA was reverse transcribed using polyT primers and the Expand Reverse Transcriptase kit (Roche) according to the manufacturer’s instructions. Real time quantitative PCR reaction was performed using a 7900HT sequence detector system (Applied Biosystems) with the SYBR Green PCR Master Mix (Applied Biosystems) according to the manufacturer’s protocol. Each reaction was performed in triplicate with 0.4 μL of the first strand cDNA in a total volume of 20 μL. The specificity of the PCR amplification was checked with a heat dissociation curve (from 60°C to 95°C). Comparative threshold values were normalized to 18S RNA internal control. The specificity of the obtained RT-PCR products was controlled on 1.8% agarose gels. Primers used for RT-PCRs of *PGI1*, *BAM1*, *BAM2*, *BAM3* and *BAM5* are listed in S2 Table.

### Confocal microscopy

Subcellular localization of GFP-tagged GBSS was performed using D-Eclipse C1 confocal microscope (NIKON, Japan) equipped with standard Ar 488 laser excitation, BA515/30 filter for green emission and BA650LP filter for red emission.

### Light and electron microscopy

Light microscopy and TEM analyses were carried out essentially as described in [105]. Briefly, small pieces (2 mm^2^) of leaves were immediately fixed by submersion in a solution of 3% glutaraldehyde (v/v) in 0.05 M sodium cacodylate buffer, pH 7.4 (3 h at 4°C, under vacuum). After fixing, the specimens were washed in a cacodylate buffer (0.05 M sodium cacodylate, 1% sucrose), three times for 30 min each at 4°C, and post-fixed with a solution of 1% osmium tetroxide in the above cacodylate buffer (overnight, 4°C). After two washes, 30 min each, at 4°C with the same cacodylate buffer, the samples were dehydrated in an ethanol series and progressively embedded in LR White resin (London Resin Co., Reading, UK). Semithin (1 μm) sections were stained with 1% (w/v) toluidine blue in aqueous 1% sodium borate for direct observation with a Zeiss Axiophot photomicroscope (Zeiss, Oberkochen, Germany). Ultrathin (70–90 nm) sections for TEM were constructed with 2% aqueous uranyl acetate and lead citrate. Observations were performed with a STEM LEO 910 electron microscope (Oberkochen, Germany) at 80 kV, equipped with a Gatan Bioscan 792 camera (Gatan, Pleasanton, CA, USA).

### Statistical analysis

The data presented are the means of three independent experiments, with 3–5 replicates for each experiment (means ± SE). The significance of differences between the control and the transgenic lines was statistically evaluated with Student’s t-test using the SPSS software. Differences were considered significant at a probability level of P<0.05. In CKs analyses, significance was determined by one-way univariate analysis of variance (ANOVA) for parametric data and Kruskal Wallis for non-parametric data, using the open source R software 2.15.1 (http://cran.r-project.org/). Multiple comparisons after ANOVA were calculated using the post hoc Tukey’s honestly significant difference (HSD) test.

## Supporting Information

S1 Fig Nucleotide sequences of complete pPGI encoding cDNAs obtained from L*er* and N92274 (*pgi1–3*) plants. (PPT)

S2 Fig Amino acid sequences deduced from the nucleotide sequences shown in S1 Fig. (PPT)

S3 Fig Iodine staining of source leaves of L*er*, *pg1–3*, Ws-2, *pgi1–2*, *aps1* and *pgm* plants. Leaves were harvested from 30 DAG plants after 12 h of illumination. Plants were cultured under LD conditions.
(EPS)

S4 Fig High expression levels of plastidial and extraplastidial β-amylase encoding genes in *pgi1* plants. RT-PCR of (A) *BAM1*, (B) *BAM2*, (C) *BAM3* and (D) *BAM5* in source leaves of WT (L*er* and Ws-2), *pgi1–2* and *pgi1–3* plants. Fully developed, source leaves were harvested from 30 DAG plants after 16 h of illumination.
(EPS)

S5 Fig Non-reducing western blot of APS1 in leaves of L*er*, *pg1–3*, Ws-2 and *pgi1–2* plants. Leaves were harvested from 30 DAG plants after 12 h of illumination. Plants were cultured under LD conditions
(EPS)

S6 Fig Respiratory CO_2_ production in darkened WT and *pgi1* leaves. Fully developed, source leaves were harvested from 30 DAG plants. Values represent the mean ± SE of determinations on five independent samples. Each sample included leaves from 3 different rosettes.
(EPS)

S7 Fig Stages to construct the *35S-PGI1* and 35S-PGI1* plasmids necessary to produce *PGI1* and PGI1* expressing plants. To obtain *PGI1* and PGI1* cDNAs, 1.5 μg RNA extracted from WT and *pgi1–3* roots was reverse transcribed using polyT primers and the Expand Reverse Transcriptase kit (Roche) according to the manufacturer’s instructions. PCR reactions were performed to generate *att*B-flanked PCR products using *PGI1* specific primers containing *att*B1 and *att*B2 recombinational cloning sites (*att*B1 primer: 5'-GGGGACAAGTTTGTACAAAAAAGCAGGCTTAAT GGCCTCTCTCTCAGGC-3'; *att*B2 primer: 5'-GGGGACCACTTTGTACAAGAAAG CTGGGTATTATGCGTACAGGTCATCCAC-3') to incorporate complete *att*B1 and *att*B2 sequences into the final PCR products.
(PPT)

S8 Fig Stages to produce the 35S-GBSS-GFP* construct. (PPT)

S1 Table Primers used to identify *pgi1–2/sex1* and *pgi1–2/gpt2* mutants by PCR. (DOC)

S2 Table Primers used in Real Time PCR. (DOC)
