# Correction: Plastidic Phosphoglucose Isomerase Is an Important Determinant of Starch Accumulation in Mesophyll Cells, Growth, Photosynthetic Capacity, and Biosynthesis of Plastidic Cytokinins in Arabidopsis

**DOI:** 10.1371/journal.pone.0126531

**Published:** 2015-04-23

**Authors:** Abdellatif Bahaji, Ángela M. Sánchez-López, Nuria De Diego, Francisco J. Muñoz, Edurne Baroja-Fernández, Jun Li, Adriana Ricarte-Bermejo, Marouane Baslam, Iker Aranjuelo, Goizeder Almagro, Jan F. Humplík, Ondřej Novák, Lukáš Spíchal, Karel Doležal, Javier Pozueta-Romero

The affiliation for the 12^th^ and 13^th^ authors is incorrect. Ondřej Novák and Lukáš Spíchal are not affiliated with #3 but with #2 Department of Chemical Biology and Genetics, Centre of the Region Haná for Biotechnological and Agricultural Research, Faculty of Science, Palacký University, Olomouc, CZ-78371, Czech Republic.

In the Funding section, the grant number from the funder Fondo Europeo de Desarrollo Regional (Spain) is listed incorrectly. The correct grant number is: BIO2013-49125-C2-1-P.
