# Correction: Correction: Plastidic Phosphoglucose Isomerase Is an Important Determinant of Starch Accumulation in Mesophyll Cells, Growth, Photosynthetic Capacity, and Biosynthesis of Plastidic Cytokinins in Arabidopsis

**DOI:** 10.1371/journal.pone.0128736

**Published:** 2015-05-15

**Authors:** The PLOS ONE Staff

There is an error in the correction published on April 23, 2015. The affiliation of Dr. Ondrej Novak is correct in the original article, and the affiliation for the 13th and 14th authors is incorrect. Lukáš Spíchal and Karel Dolezal are not affiliated with #3 but with #2 Department of Chemical Biology and Genetics, Centre of the Region Haná for Biotechnological and Agricultural Research, Faculty of Science, Palacký University, Olomouc, CZ-78371, Czech Republic. The publisher apologizes for the error.

## References

[1] Bahaji A, Sánchez-López ÁM, De Diego N, Muñoz FJ, Baroja-Fernández E, Li J, et al (2015) Plastidic Phosphoglucose Isomerase Is an Important Determinant of Starch Accumulation in Mesophyll Cells, Growth, Photosynthetic Capacity, and Biosynthesis of Plastidic Cytokinins in Arabidopsis. PLoS ONE 10(3): e0119641 doi:10.1371/journal.pone.0119641 25811607 10.1371/journal.pone.0119641 PMC4374969

[2] Bahaji A, Sánchez-López ÁM, De Diego N, Muñoz FJ, Baroja-Fernández E, Li J, et al (2015) Correction: Plastidic Phosphoglucose Isomerase Is an Important Determinant of Starch Accumulation in Mesophyll Cells, Growth, Photosynthetic Capacity, and Biosynthesis of Plastidic Cytokinins in Arabidopsis. PLoS ONE 10(4): e0126531 doi:10.1371/journal.pone.0126531 25906238 10.1371/journal.pone.0126531 PMC4408045

