# Supplementary material for: Plastidic Phosphoglucose Isomerase Is an Important Determinant of Starch Accumulation in Mesophyll Cells, Growth, Photosynthetic Capacity, and Biosynthesis of Plastidic Cytokinins in Arabidopsis
Source: PLoS One. 2015 Mar 26;10(3):e0119641. doi: 10.1371/journal.pone.0119641 (PMC4374969; doi:10.1371/journal.pone.0119641)
Supplement: S1 Fig — (PPT) [file pone.0119641.s001.ppt]

## Slide 1
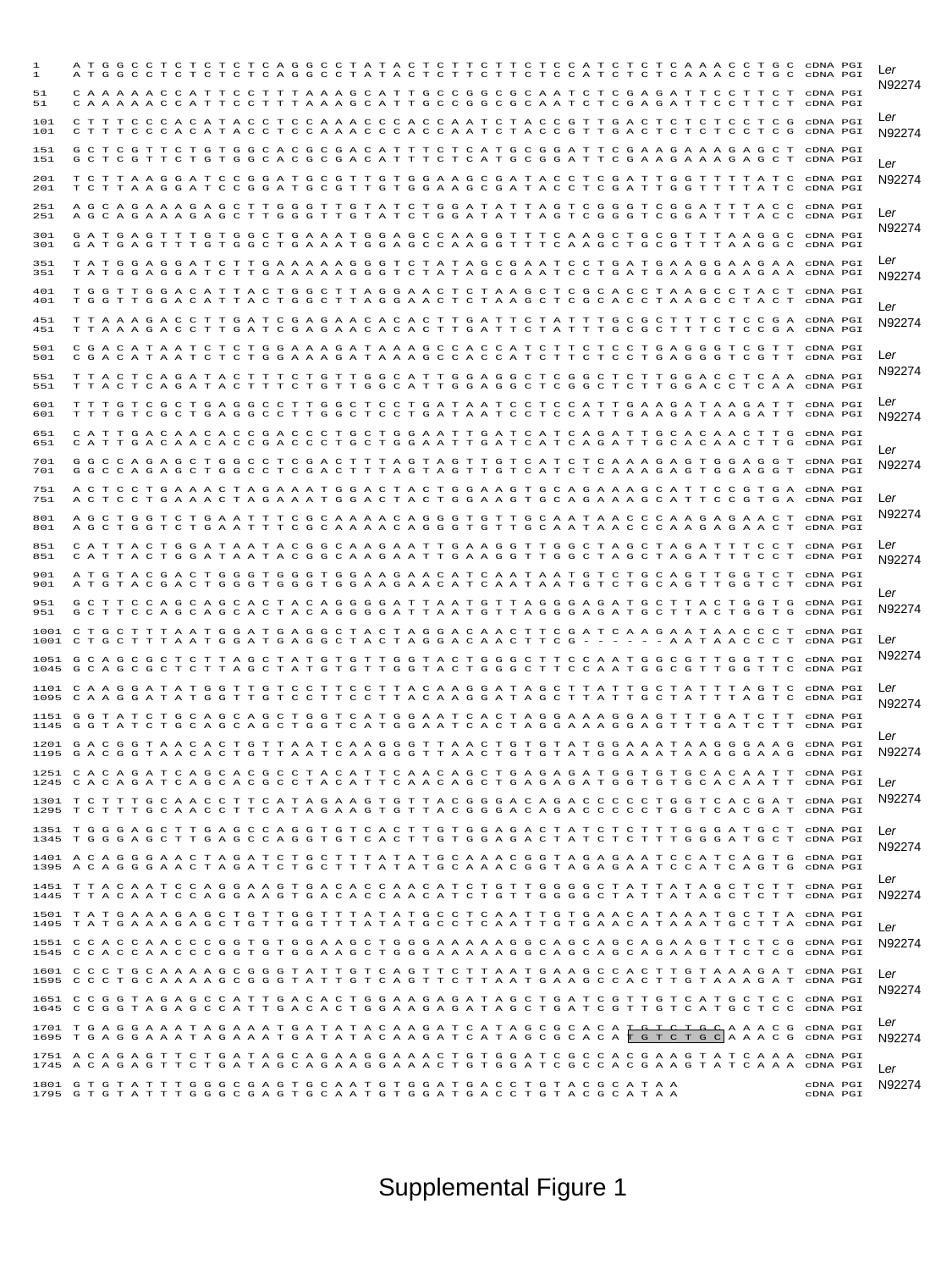

Ler
N92274
Ler
N92274
Ler
N92274
Ler
N92274
Ler
N92274
Ler
N92274
Ler
N92274
Ler
N92274
Ler
N92274
Ler
N92274
Ler
N92274
Ler
N92274
Ler
N92274
Ler
N92274
Ler
N92274
Ler
N92274
Ler
N92274
Ler
N92274
Ler
N92274
Ler
N92274
Ler
N92274
Ler
N92274
Supplemental Figure 1

## Slide 2
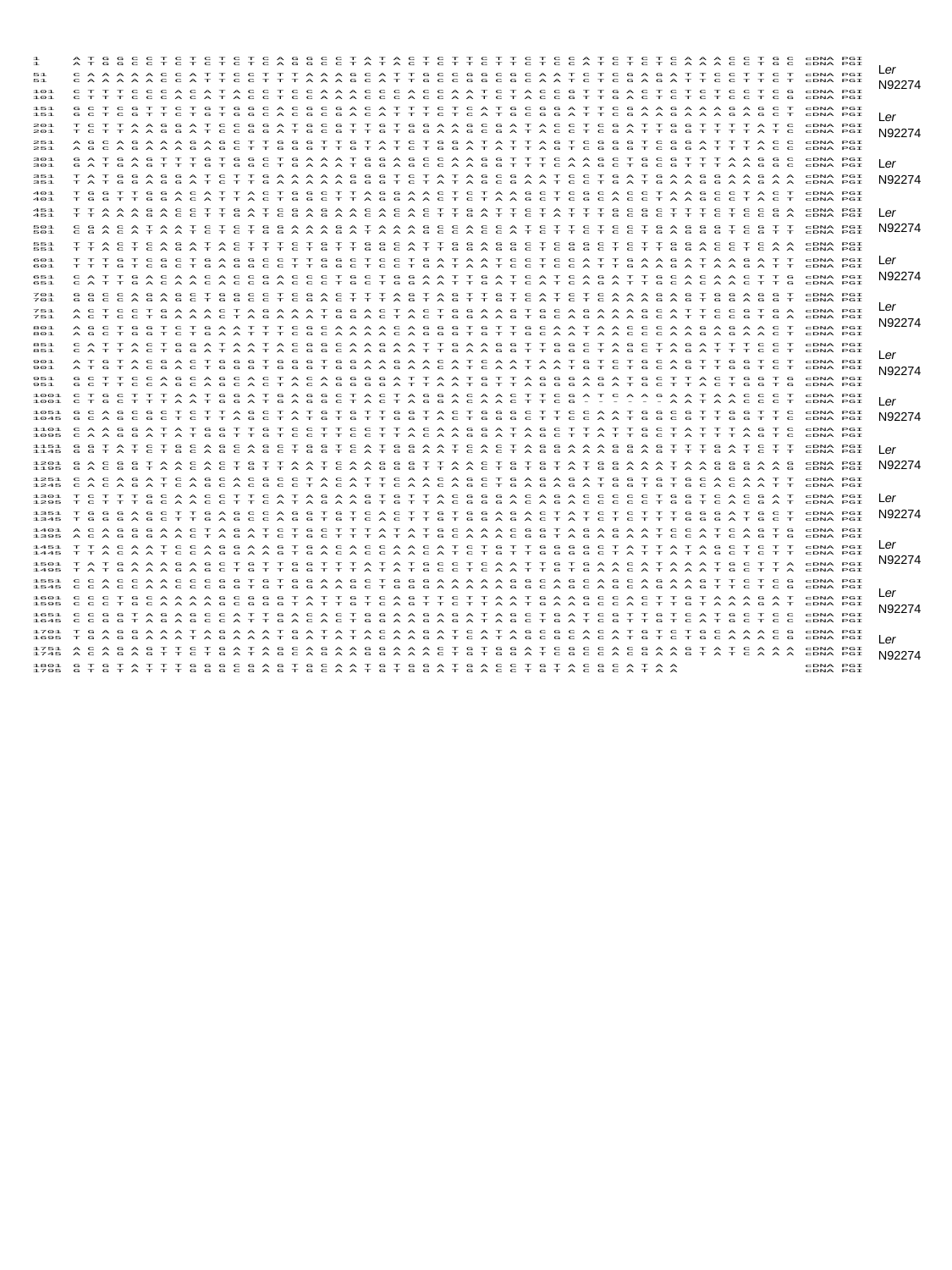

Ler
N92274
Ler
N92274
Ler
N92274
Ler
N92274
Ler
N92274
Ler
N92274
Ler
N92274
Ler
N92274
Ler
N92274
Ler
N92274
Ler
N92274
Ler
N92274
Ler
N92274
