# Supplementary figures and images for: Plastidic Phosphoglucose Isomerase Is an Important Determinant of Starch Accumulation in Mesophyll Cells, Growth, Photosynthetic Capacity, and Biosynthesis of Plastidic Cytokinins in Arabidopsis
Source: PLoS One. 2015 Mar 26;10(3):e0119641. doi: 10.1371/journal.pone.0119641 (PMC4374969; doi:10.1371/journal.pone.0119641)

## Slide 1
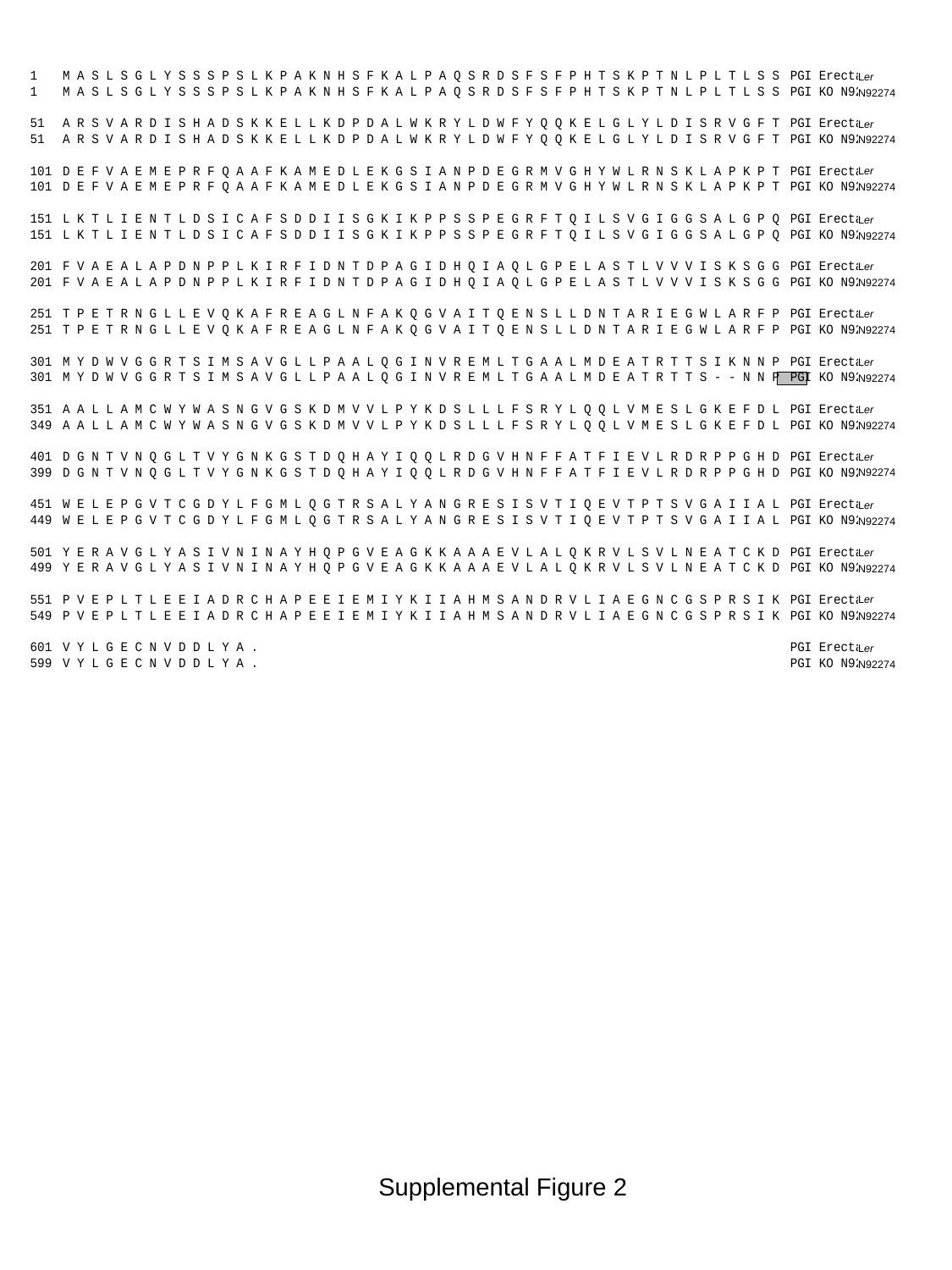

Ler
N92274
Ler
N92274
Ler
N92274
Ler
N92274
Ler
N92274
Ler
N92274
Ler
N92274
Ler
N92274
Ler
N92274
Ler
N92274
Ler
N92274
Ler
N92274
Ler
N92274
Supplemental Figure 2

Supplement: S2 Fig — (PPT) [file pone.0119641.s002.ppt]
