# Supplementary material for: Plastidic Phosphoglucose Isomerase Is an Important Determinant of Starch Accumulation in Mesophyll Cells, Growth, Photosynthetic Capacity, and Biosynthesis of Plastidic Cytokinins in Arabidopsis
Source: PLoS One. 2015 Mar 26;10(3):e0119641. doi: 10.1371/journal.pone.0119641 (PMC4374969; doi:10.1371/journal.pone.0119641)
Supplement: S7 Fig — To obtain PGI1 and PGI1* cDNAs, 1.5 μg RNA extracted from WT and pgi1–3 roots was reverse transcribed using polyT primers and the Expand Reverse Transcriptase kit (Roche) according to the manufacturer’s instructions. PCR reactions were performed to generate attB-flanked PCR products using PGI1 specific primers containing attB1 and attB2 recombinational cloning sites (attB1 primer: 5'-GGGGACAAGTTTGTACAAAAAAGCAGGCTTAAT GGCCTCTCTCTCAGGC-3'; attB2 primer: 5'-GGGGACCACTTTGTACAAGAAAG CTGGGTATTATGCGTACAGGTCATCCAC-3') to incorporate complete attB1 and attB2 sequences into the final PCR products. (PPT) [file pone.0119641.s007.ppt]

## Slide 1
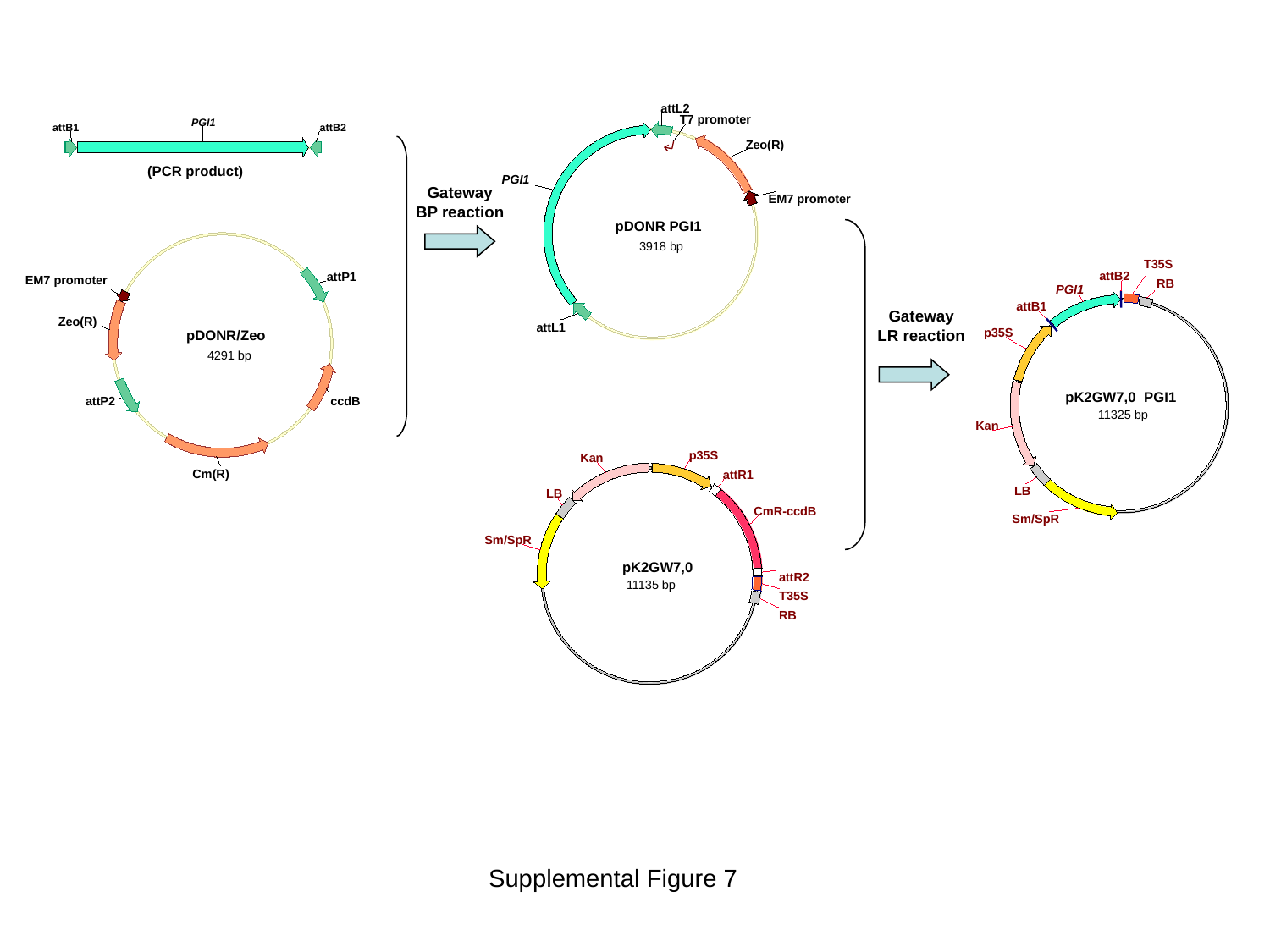

attL2
T7 promoter
Zeo(R)
PGI1
EM7 promoter
pDONR PGI1
3918 bp
attL1
PGI1
attB1
attB2
(PCR product)
Gateway
BP reaction
attP1
EM7 promoter
Zeo(R)
pDONR/Zeo
4291 bp
attP2
ccdB
Cm(R)
T35S
attB2
RB
PGI1
attB1
p35S
pK2GW7,0 PGI1
11325 bp
Kan
LB
Sm/SpR
Gateway
LR reaction
p35S
Kan
attR1
LB
CmR-ccdB
Sm/SpR
pK2GW7,0
attR2
11135 bp
T35S
RB
Supplemental Figure 7

## Slide 2
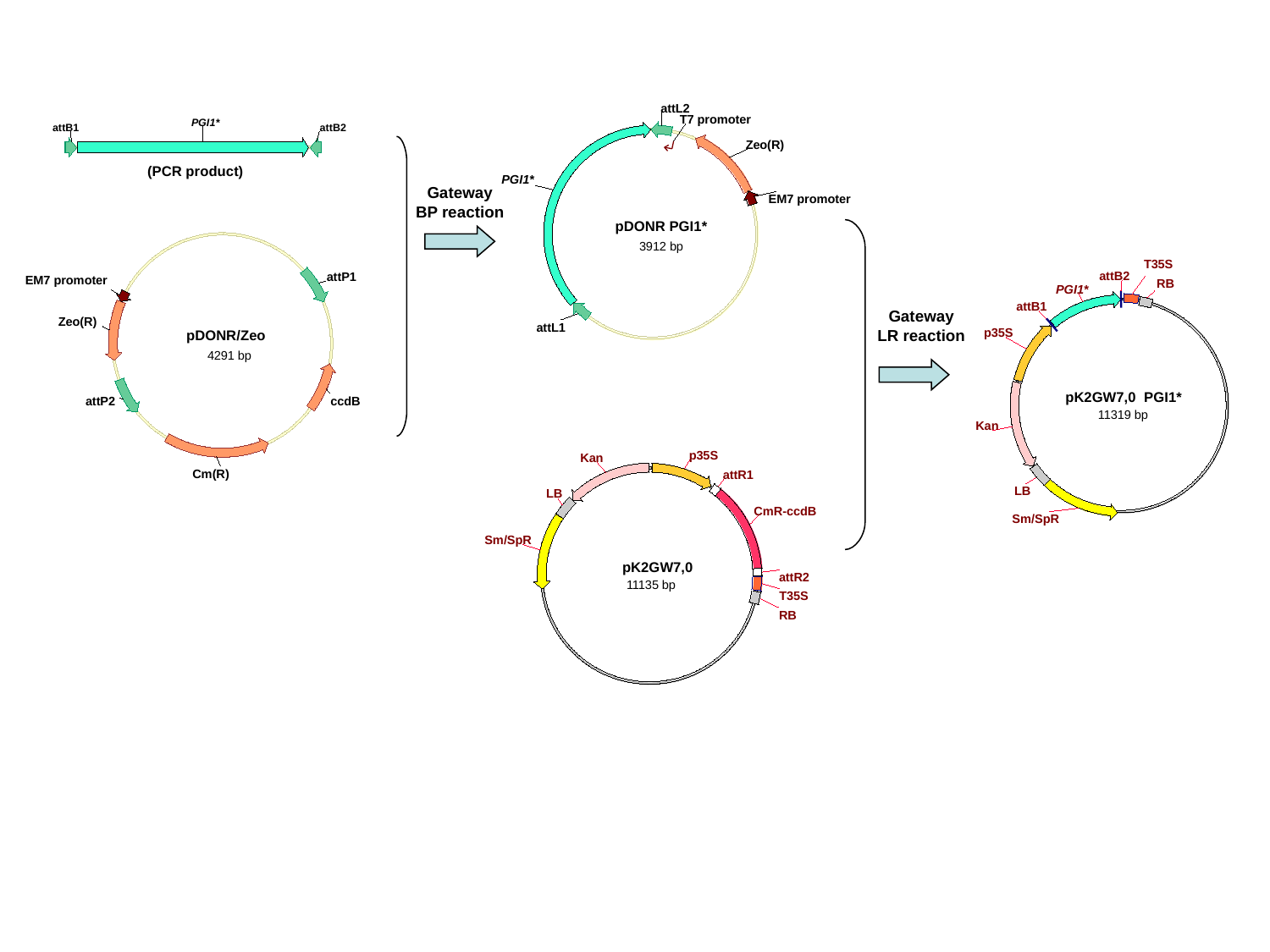

attL2
T7 promoter
Zeo(R)
PGI1*
EM7 promoter
pDONR PGI1*
3912 bp
attL1
PGI1*
attB1
attB2
(PCR product)
Gateway
BP reaction
attP1
EM7 promoter
Zeo(R)
pDONR/Zeo
4291 bp
attP2
ccdB
Cm(R)
T35S
attB2
RB
PGI1*
attB1
p35S
pK2GW7,0 PGI1*
11319 bp
Kan
LB
Sm/SpR
Gateway
LR reaction
p35S
Kan
attR1
LB
CmR-ccdB
Sm/SpR
pK2GW7,0
attR2
11135 bp
T35S
RB
