# Supplementary material for: Plastidic Phosphoglucose Isomerase Is an Important Determinant of Starch Accumulation in Mesophyll Cells, Growth, Photosynthetic Capacity, and Biosynthesis of Plastidic Cytokinins in Arabidopsis
Source: PLoS One. 2015 Mar 26;10(3):e0119641. doi: 10.1371/journal.pone.0119641 (PMC4374969; doi:10.1371/journal.pone.0119641)
Supplement: S8 Fig — (PPT) [file pone.0119641.s008.ppt]

## Slide 1
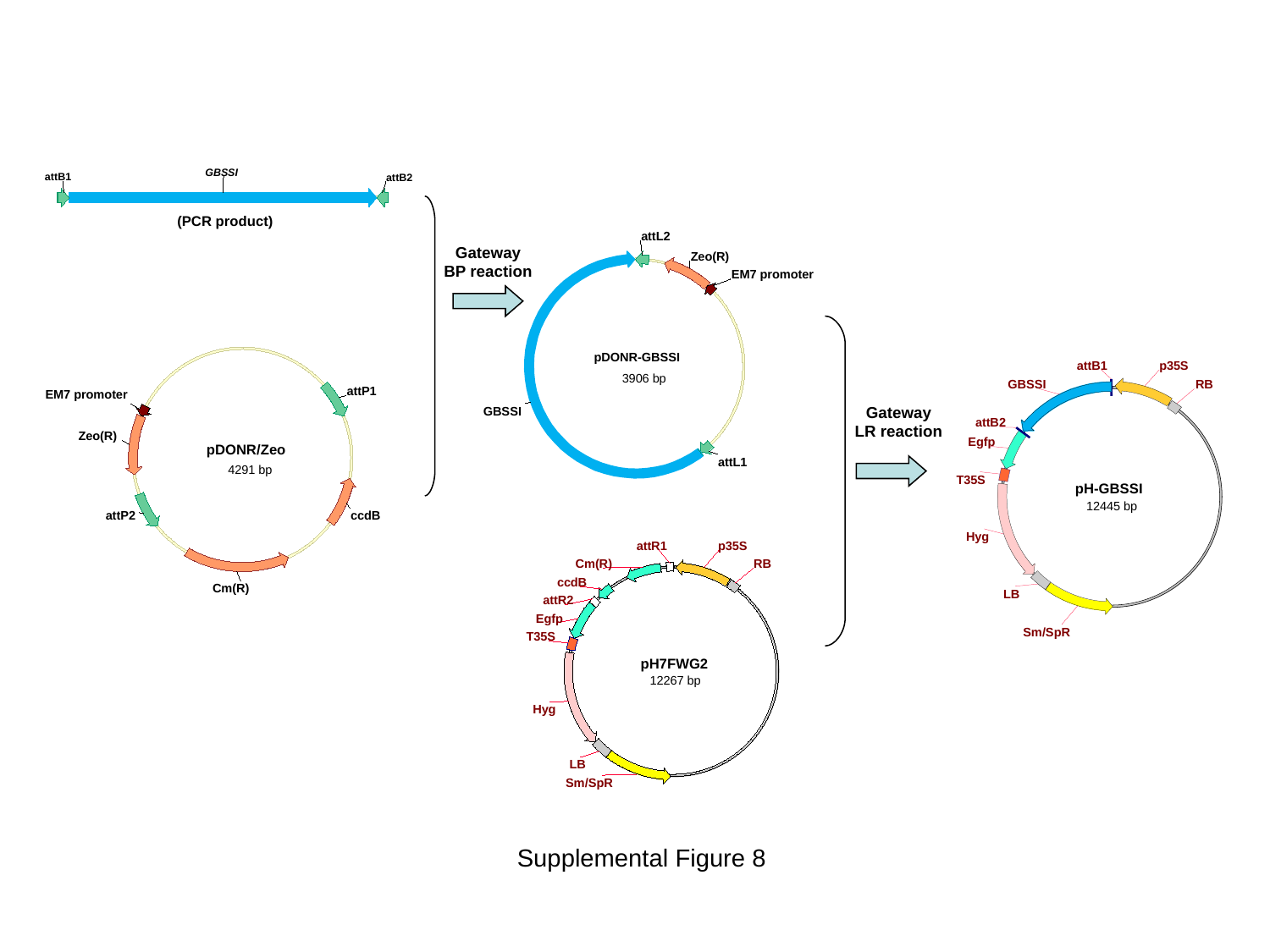

GBSSI
attB1
attB2
(PCR product)
attL2
Zeo(R)
EM7 promoter
pDONR-GBSSI
3906 bp
GBSSI
attL1
Gateway
BP reaction
attB1
p35S
GBSSI
RB
attB2
Egfp
T35S
pH-GBSSI
12445 bp
Hyg
LB
Sm/SpR
attP1
EM7 promoter
Gateway
LR reaction
Zeo(R)
pDONR/Zeo
4291 bp
attP2
ccdB
attR1
p35S
Cm(R)
RB
ccdB
attR2
Egfp
T35S
pH7FWG2
12267 bp
Hyg
LB
Sm/SpR
Cm(R)
Supplemental Figure 8
