# Supplementary material for: Plastidic Phosphoglucose Isomerase Is an Important Determinant of Starch Accumulation in Mesophyll Cells, Growth, Photosynthetic Capacity, and Biosynthesis of Plastidic Cytokinins in Arabidopsis
Source: PLoS One. 2015 Mar 26;10(3):e0119641. doi: 10.1371/journal.pone.0119641 (PMC4374969; doi:10.1371/journal.pone.0119641)
Supplement: S1 Table — (DOC) [file pone.0119641.s009.doc]

## Supplemental Table 1. Primers used to identify *pgi1-2/sex1* and *pgi1-2/gpt2* mutants by PCR

| **Mutant** | **Designation** | **Sequence** |
| --- | --- | --- |
| *pgi1-2* | Forward | 5’-TATACTCTTCTTCTCCATCTCTCAAAC-3’ |
|  | Reverse | 5’-CTTTTAATCAGAAAAACCTAAGAGAGG-3’ |
|  | T-DNA | 5’-CATTTTATAATAACGCTGCGGACATCTAC-3’ |
| *sex1* | Forward | 5’-GTCAGTCTATCCTGCGCTTTG-3’ |
|  | Reverse | 5’-TCCGGTATGACAAGTCGAATC-3’ |
|  | T-DNA | 5’-GCGTGGACCGCTTGCTGCAACT-3’ |
| *gpt2* | Forward | 5’-CTTCATGGGAGAGACTTTCCC-3’ |
|  | Reverse | 5’-TGATCTCACCGGAATGTTCTC-3’ |
|  | T-DNA | 5’-cccatttggacgtgaatgtagacac-3’ |
