# Supplementary material for: Plastidic Phosphoglucose Isomerase Is an Important Determinant of Starch Accumulation in Mesophyll Cells, Growth, Photosynthetic Capacity, and Biosynthesis of Plastidic Cytokinins in Arabidopsis
Source: PLoS One. 2015 Mar 26;10(3):e0119641. doi: 10.1371/journal.pone.0119641 (PMC4374969; doi:10.1371/journal.pone.0119641)
Supplement: S2 Table — (DOC) [file pone.0119641.s010.doc]

**Supplemental Table 2.** Primers used in Real Time PCR

| **Gene** | **Direction** | **Sequence** |
| --- | --- | --- |
| 18S RNA | Forward | 5´-gggcattcgtatttcatagtcagag-3´ |
| At3g41768 | Reverse | 5´-cggttcttgattaatgaaaacatcct-3´ |
|  |  |  |
| pPGI | Forward | 5´-GGGATTAATGTTAGGGAGATGC-3´ |
| At4g24620 | Reverse | 5´-TGTTACCGTCAAGATCAAACTC-3´ |
|  |  |  |
| BAM1 | Forward | 5´-cttgatcaaaacaatgagggag-3´ |
| At3g23920 | Reverse | 5´-cttcccgacatctatgtgag-3´ |
|  |  |  |
| BAM2 | Forward | 5´-accgatcctgatggtcgcc-3´ |
| At4g00490 | Reverse | 5´-ctcttgtgtccctggagcc-3´ |
|  |  |  |
| BAM3 | Forward | 5´-gattctggaaatgggttaacc-3´ |
| At4g17090 | Reverse | 5´-gtcacttccagttgtgtcttc-3´ |
|  |  |  |
| BAM5 | Forward | 5´-CACTACGGCATTCTCAACTTC-3’ |
| At4g15210 | Reverse | 5´-CCTTTGGCTCCATAGGTCTC-3’ |
